# Integrated Transcriptomic Analysis of S100A8/A9 as a Key Biomarker and Therapeutic Target in Sepsis Pathogenesis and AI Drug Repurposing

**DOI:** 10.3390/ijms262211186

**Published:** 2025-11-19

**Authors:** Kirtan Dave, Alejandro Pazos-García, Natia Tamarashvili, Jose Vázquez-Naya, Cristian R. Munteanu

**Affiliations:** 1 Parul Institute of Applied Sciences, Department of Life Sciences, Parul University, Vadodara 391760, India; 2 Bioinformatics Laboratory, Research & Development Cell, Parul University, Vadodara 391760, India; 3 RNASA, CITIC, Computer Science Faculty, University of A Coruña, 15071 A Coruña, Spain; alejandro.pazos.garcia@udc.es (A.P.-G.); jose.manuel.vazquez.naya@udc.es (J.V.-N.); 4 School of Medicine, New Vision University, 0159 Tbilisi, Georgia; ntamarashvili@newvision.ge

**Keywords:** S100A8/A9, TLR4, scRNA-seq, AI-driven drug repurposing, NAMPT, bioinformatics

## Abstract

Sepsis is a life-threatening condition driven by a dysregulated immune response, leading to systemic inflammation and multi-organ failure. Among the key molecular regulators, S100A8/A9 has emerged as a critical damage-associated molecular pattern (DAMP) protein, amplifying pro-inflammatory signaling via the Toll-like receptor 4 (TLR4) and receptor for advanced glycation end products (RAGE) pathways. Elevated S100A8/A9 levels correlate with disease severity, making it a promising biomarker and therapeutic target. To unravel the role of S100A8/A9 in sepsis, we integrate scRNA-seq and RNA-seq approaches. scRNA-seq enables cell-type-specific resolution of immune responses, uncovering cellular heterogeneity, state transitions, and inflammatory pathways at the single-cell level. In contrast, RNA-seq provides a comprehensive view of global transcriptomic alterations, allowing robust statistical analysis of differentially expressed genes. The integration of both approaches enables precise deconvolution of immune cell contributions, validation of cell-specific markers, and identification of potential therapeutic targets. Our findings highlight the S100A8/A9-driven inflammatory cascade, its impact on immune cell interactions, and its potential as a diagnostic and prognostic biomarker in sepsis. Eight protein targets resulted from the integrative transcriptomics studies (corresponding to S100A8, S100A9, S100A6, NAMPT, FTH1, B2M, KLF6 and SRGN) have been used to predict interaction affinities with 2958 ChEMBL approved drugs, by using a pre-trained AI models (PLAPT) in order to point directions on drug repurposing in sepsis. The strongest predicted interactions have been confirmed with molecular docking and molecular dynamics analysis. This study underscores the power of combining high-throughput transcriptomics to advance our understanding of sepsis pathophysiology and develop precision medicine strategies.

## 1. Introduction

Sepsis is a critical illness characterized by a dysregulated immune response to infection, often leading to multiple organ failure. Septic shock is a major contributor to mortality among critically ill patients worldwide, with a 30-day mortality rate of approximately 34.7% [1]. Despite therapeutic advancements like antibiotics and supportive care, sepsis remains a global health concern, accounting for over 47 million cases and more than 11 million fatalities each year [2]. Current treatments often fail to address the fundamental immune system disturbances and irregularities in the host’s response to infection [3], highlighting the urgent need for improved preventative, diagnostic, and therapeutic approaches [4]. The pathophysiology of sepsis is predominantly influenced by an exaggerated inflammatory response [5]. S100A8/A9 (calprotectin), a key damage-associated molecular pattern (DAMP) protein, is significantly elevated in sepsis and plays a crucial role in amplifying IL-17-mediated inflammation through multiple interconnected mechanisms.

First, S100A8/A9 binds to Toll-like receptor 4 (TLR4) and the receptor for advanced glycation products (RAGE), triggering NF-κB and MAPK activation. This leads to increased production of pro-inflammatory cytokines such as IL-6, TNF-α, and IL-1β, which further enhance IL-17 expression. Additionally, S100A8/A9 promotes the secretion of IL-23 and IL-1β by antigen-presenting cells (APCs), crucial signals for Th17 differentiation. Simultaneously, it suppresses regulatory T cells (Tregs), reducing IL-17 inhibition and allowing sustained inflammation. Furthermore, the activation of pathogen-associated molecular patterns (PAMPs) stimulates pattern recognition receptors (PRRs), resulting in a vigorous innate immune response [5]. The interaction between S100A8/A9 and IL-17 creates a self-sustaining inflammatory loop. IL-17 signaling recruits neutrophils and macrophages, leading to further S100A8/A9 release, thereby reinforcing the inflammatory response. This synergy extends to the induction of neutrophil extracellular traps (NETs), which contribute to excessive reactive oxygen species (ROS) production, endothelial dysfunction, and vascular damage, ultimately leading to multi-organ failure in sepsis [6]. Moreover, S100A8/A9 shifts immune cells toward a pro-inflammatory metabolic state, sustaining IL-17 production. Epigenetic modifications, such as histone acetylation, further prolong IL-17 transcription, exacerbating immune hyperactivation even after pathogen clearance [7].

Given its critical role in sepsis, targeting S100A8/A9-TLR4/RAGE signaling presents a potential therapeutic strategy to disrupt the IL-17-driven inflammatory cascade. Inhibitors targeting S100A8/A9 or IL-17, such as anti-S100A8/A9 antibodies or IL-17 inhibitors (e.g., Secukinumab), may help mitigate immune dysregulation and inflammation in sepsis, offering promising avenues for improved patient outcomes. However, sepsis often exacerbates hospital-acquired infections, viral reactivation, and immunological impairment, leading to unfavorable patient outcomes [8]. Despite extensive research, there are currently few specific treatments for sepsis. This lack of specific treatments stems from the intricate interactions between numerous cells and molecules that drive sepsis, which are challenging to fully comprehend in a lab setting by focusing on a single component at a time [9].

This highlights the value of a multiomics approach for achieving a thorough understanding of S100A8/A9 function [5,6]. As calcium-binding and pro-inflammatory proteins, S100A8/A9 regulate critical immunological functions and are heavily involved in the inflammatory response. This underscores the importance of studying S100A8/A9 in the context of disease dynamics [10,11].

Advances in transcriptomics, particularly RNA sequencing (RNA-seq), have provided powerful tools to unravel the molecular underpinnings of sepsis [12]. RNA-seq has been widely applied to identify biomarkers for sepsis diagnosis and prognosis by analyzing changes in gene expression. However, this approach evaluates the “average” expression across all cells, making it less effective at detecting cellular heterogeneity [13]. scRNA-seq overcomes this limitation by offering granular insights into the transcriptomic diversity of individual cells [14], enabling the identification of critical immune and inflammatory cell subsets involved in sepsis progression [15].

In this study, we integrated scRNA-seq and RNA-seq data to investigate changes in immune cell type fractions, the expression pattern of cell death-related genes, and differentially expressed genes. This work examines the activation of the IL-17 pathway [16] induced by S100A8/A9, emphasizing its pro-inflammatory function in sepsis and multi-organ failure [17]. This pathway, recognized for its role in chronic inflammation, may offer novel treatment strategies for the management of sepsis and its related consequences [18].

Additionally, studies on potential drugs targeting S100A8/A9 proteins using Machine Learning models are limited [19]. Therefore, our work calculates the interaction affinities between sepsis-linked targets and approved drugs using a pre-trained AI model, and confirms the best interactions with molecular docking and molecular dynamics. These results represent the first step in future drug repurposing possibilities [20].

## 2. Results

### 2.1. Patient Cohort Description

To characterize immune dysregulation and transcriptional alterations in sepsis, we analyzed single-cell RNA-seq (scRNA-seq, GSE167363) and bulk RNA-seq (GSE196117) datasets. The scRNA-seq cohort consisted of peripheral blood mononuclear cells (PBMCs) from 10 sepsis patients, including both survivors and non-survivors, and 2 healthy controls, enabling the identification of cell-type-specific transcriptional patterns. The bulk RNA-seq cohort comprised whole-blood RNA from 33 sepsis patients and 7 healthy controls, providing a system-level view of gene expression.

### 2.2. Identification of Cell Subtypes in scRNA-Seq Data

**Figure 1:**
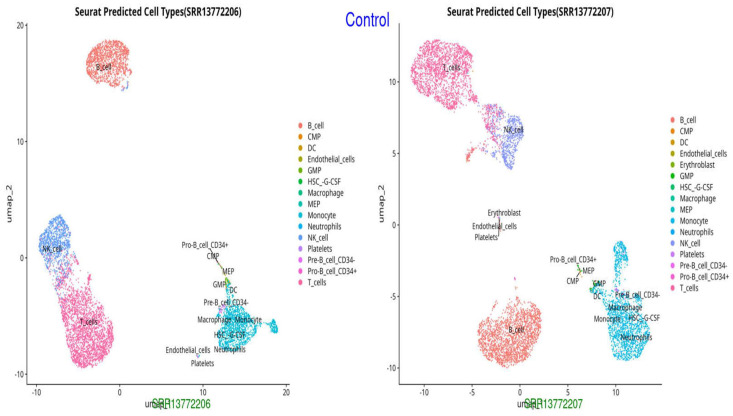
UMAP representation of cell types in the Control group, showing normal distribution and clustering of immune cells (monocytes, neutrophils, endothelial cells, and T cells).

**Figure 2:**
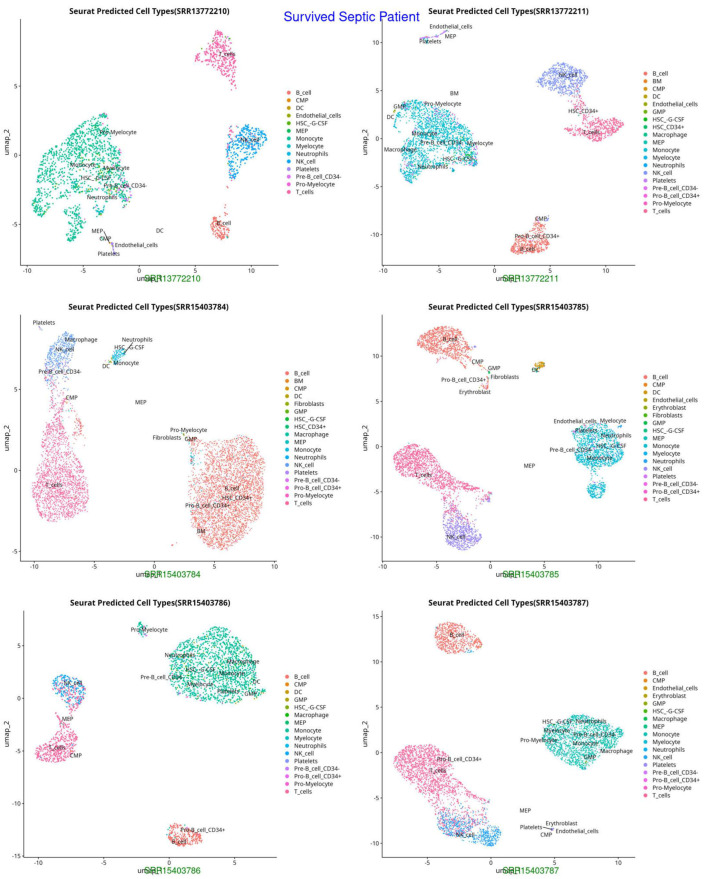
UMAP representation of cell types in Survived Septic Patients, highlighting shifts in immune cell populations post-sepsis recovery (HSC-G-CSF, macrophages, neutrophils, and activated T cells, indicating immune recovery and adaptation).

**Figure 3:**
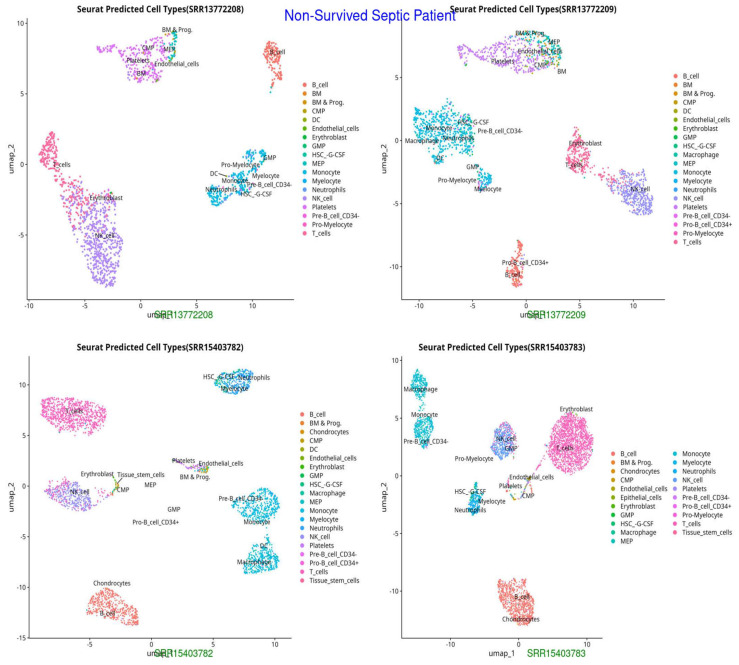
UMAP representation of cell types in Non-Survived Septic Patients, indicating distinct clustering patterns and potential immune dysregulation (HSC-G-CSF, pro-myelocytes), excessive neutrophil activation, and potential endothelial dysfunction, reflecting immune failure and systemic inflammation.

We initially excluded ineligible cells based on the following standardized quality-control criteria: total RNA counts (nCount) > 1000, detected gene features (nFeature) > 750, and mitochondrial gene percentage < 5%. Genes were retained if expressed in at least 13 cells, yielding a final dataset of 36,866 cells and 12,661 genes for subsequent analysis. From these, 2888 highly variable genes were identified. The top 10 principal components were then used for UMAP dimensionality reduction (Figure 1, Figure 2 and Figure 3). Thirteen distinct cell groups were identified, including B cells, T cells, monocytes, myeloid cells, activated macrophages, neutrophils, CD4+ and CD8+ T cells, regulatory T cells, inflammatory monocytes, highly activated T cells, and dendritic cells. Nine cell types were classified by identifying marker genes using CellMarker. These findings highlight the diversity of immune cell types in the scRNA-seq data.

The UMAP and t-SNE projections demonstrate divergent immunological dynamics among the three groups: Control, Survived Septic Patients, and Non-Survived Septic Patients. The control group exhibits a balanced and homeostatic immune state characterized by various immune cell types, including B cells, T cells, monocytes, and myeloid cells, with low ligand–receptor interactions, signifying a stable environment with restricted activation. Conversely, septic patients who survived display predominant numbers of activated macrophages, neutrophils, CD4+ and CD8+ T cells, and regulatory T cells, indicating an adaptive immunological response and evidence of immune resolution. Augmented ligand–receptor interactions among regulatory T cells, macrophages, and dendritic cells underscore collaborative efforts for tissue healing and immunological equilibrium. In non-survived septic patients, immunological dysregulation is apparent, characterized by predominant populations of neutrophils, inflammatory monocytes, highly activated T cells, and dendritic cells, which contribute to hyperactivation, cytokine storms, and severe inflammation. Intense ligand–receptor interactions, especially between neutrophils and monocytes, highlight a pro-inflammatory condition that leads to immunological fatigue and multi-organ failure.

### 2.3. Analysis of Signaling Communication Probability Between Two Cell Groups

We utilized the scRNA-seq data and the “CellChat” R package, Version 1.5.0 to examine cellular communication. Cell communication network maps and interaction heatmaps demonstrated a significant rise in both the quantity and intensity of contacts between platelets and diverse cells in the sepsis group relative to the control group. Examination of ligand–receptors revealed elevated interaction between platelets and various cell types in sepsis compared to the control group. Further investigation of ligand–receptor interactions and gene expression profiles uncovered unique signaling communication patterns among immune cell types in septic patients.

In the control group, monocytes, T cells, and B cells maintained the immune system’s normal function and homeostasis with limited interactions. Dendritic cells, on the other hand, facilitated antigen presentation and immune system activation with very little ligand–receptor engagement. In septic patients who survived, neutrophils and monocytes improved immune responses and tissue repair through moderate ligand–receptor interactions. Concurrently, NK cells and T cells facilitated the proliferation and differentiation of immune cells, which aided patient recovery.

In non-surviving septic patients, monocytes and macrophages exhibited hyperactivation, resulting in a cytokine storm characterized by elevated ligand–receptor interactions indicative of severe immune activation. Concurrently, neutrophils and dendritic cells displayed vigorous immune responses that culminated in immune exhaustion and organ failure, with excessive ligand–receptor interactions indicating immune dysregulation. The varying communication probabilities suggest that certain immune cell contacts facilitate healing, while others exacerbate the pathogenic overactivation of the immune system in septic settings, as illustrated in Supplementary Figures S1–S6. These cells are critical contributors to the excessive immune response and immune exhaustion seen in non-surviving septic patients, highlighting their role in immune dysregulation during late-stage sepsis.

The immune response to infection progresses through two distinct phases: an inflammatory phase marked by increased cytokine production and an immunosuppressive phase characterized by diminished immunological signaling. The control group, comprising healthy individuals, exhibits orderly and efficient cell communication, with interactions among T cells, neutrophils, monocytes, fibroblasts, and endothelial cells, signifying a balanced immune system, undamaged blood vessels, and tissue homeostasis.

Conversely, the Survived group, comprising recovered sepsis patients, exhibits enhanced communication through improved signaling between T-cells and monocytes, promoting regulated inflammation and vascular repair, perhaps mediated by VEGF or FGF signaling. This group demonstrates moderate network complexity, signifying a resolution of hyper-inflammation and effective immune control.

The non-survived group, indicative of fatal sepsis cases, exhibits impaired communication networks characterized by increased interactions among neutrophils, monocytes, and T-cells, indicating a cytokine storm propelled by hyper-inflammatory pathways. The failure of endothelial connections worsens vascular dysfunction and capillary leakage, while redundant immunological pathways indicate that the immune system is exhausted and incapable of shifting from inflammation to resolution. Heatmaps illustrating the degree of intercellular communication demonstrate the differences among the three groups. The control group has low interaction intensity, indicating immunological homeostasis, with T-cells, macrophages, and endothelial cells preserving immune surveillance and tissue integrity. The Survived group demonstrates heightened interaction intensity, signifying adaptive immune responses characterized by balanced pro-inflammatory and anti-inflammatory signals, facilitated by substances such as IL-10 and TGF-β, alongside active tissue healing processes. Conversely, the non-survived group has intense, disorganized communication, characterized by hyperactive neutrophils and macrophages that secrete inflammatory cytokines such as IL-6, TNF-α, and IL-1β, leading to systemic inflammation and multi-organ failure. Endothelial dysfunction, vascular permeability, and microthrombosis intensify the condition. These findings underscore the essential function of intercellular communication in sepsis progression and outcomes, identifying critical molecular regulators such as NF-κB and TLR signaling as prospective therapeutic targets. Comprehending these patterns provides information for forecasting sepsis severity and customizing therapy to enhance patient outcomes.

### 2.4. Identification and Functional Enrichment Analysis of DEGs in the RNA-Seq Data

**Figure 4:**
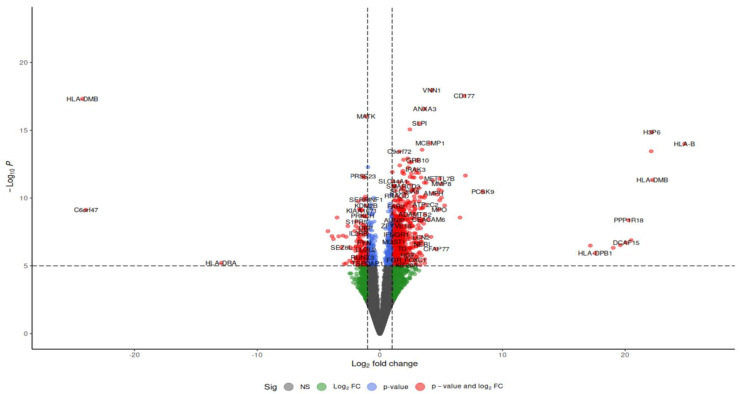
Volcano plots of the differential gene expression data from GSE196117. In the volcano plots, the red points show up-regulated genes (log2FC  ≥  0.5 and adjusted *p*-value  <  0.05). The Venn map of the differentially expressed genes, however, shows the up-regulated genes as red dots.

Differential expression between sepsis and control samples was determined by comparing gene expression levels in septic patients and healthy controls. Analysis was performed using DESeq239 with an adjusted *p*-value < 0.05 in R Studio. Upon mapping genomic reads from sepsis patients’ immune cells, including macrophages, neutrophils, and T cells, along with endothelial cells from heart and vascular tissues, heatmaps of differentially expressed genes demonstrated distinct clustering based on conditions (sepsis or control) and transcriptomic profiles. A volcano plot, depicted in Figure 4, allows for the visualization of expression changes and their significance throughout the complete gene set. Each gene is represented as a point in a two-dimensional space of statistical significance (*p*-value or FDR) versus log fold change.

**Figure 5:**
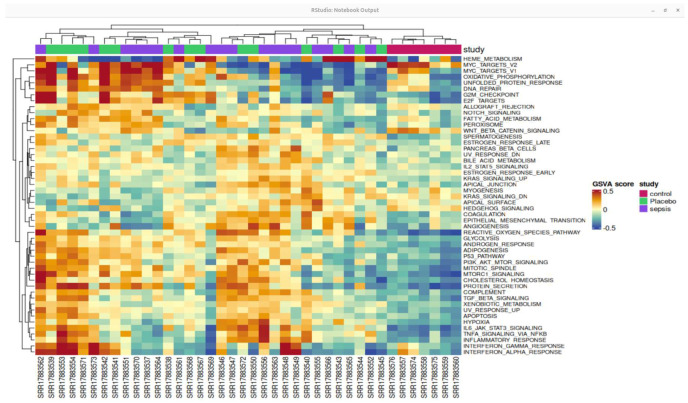
Heat map of the top differentially expressed genes based on GSE196117. The color intensity suggests the higher to lower expression pathway and pathway enrichment analysis.

A heatmap of genes with differential expression is shown in Figure 5, providing a thorough illustration of expression patterns. Hierarchical clustering was performed on both columns (samples) and rows (genes) to identify expression differences between septic and control samples. This analysis provides insight into the molecular signatures of sepsis progression, uncovering novel biomarkers and potential therapeutic targets for sepsis-associated immune dysregulation.

#### Gene Ontology of Genes and Genome Pathway Analyses

**Figure 6:**
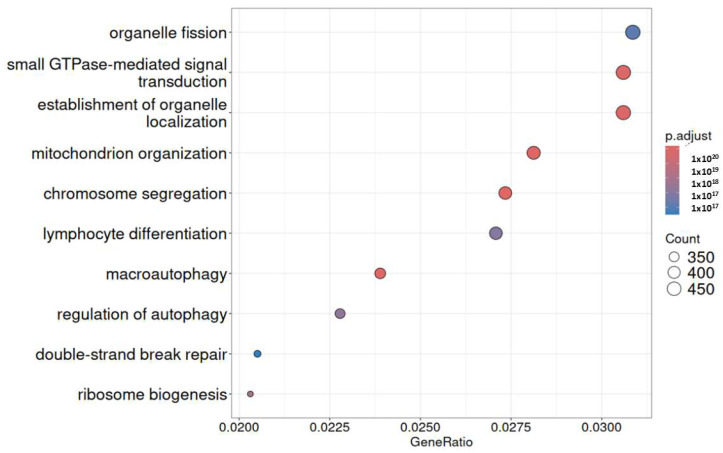
Analysis of KEGG pathway enrichment. The gene ratio is displayed on the x-axis, while the GO category is displayed on the y-axis. The size of the circle represents the number of genes included in the relevant pathway, and the significance of enrichment progressively grows from blue to red.

In all three investigations, 2122 genes were found to be up-regulated in common by a differential expression analysis among infected cardiac cells. The Venn diagram suggested that more genes were up-regulated in common among different groups. Together, GO enrichment analysis was conducted for these three gene categories, revealing that genes were strongly linked to infected cells and had a significant role in the network regulated by NF-κB signaling. Biological processes frequently include the regulation of small GTPase-mediated signal transduction, as shown by nine GO annotations. Figure 6 represents the top functional category derived from elevated genes according to KEGG analysis. It displays that one of the main mechanisms controlling inflammation is the nuclear factor-kappa B (NF-κB) pathway.

### 2.5. Eight DEGs Were Identified as Diagnostic Genes for Sepsis

**Figure 7:**
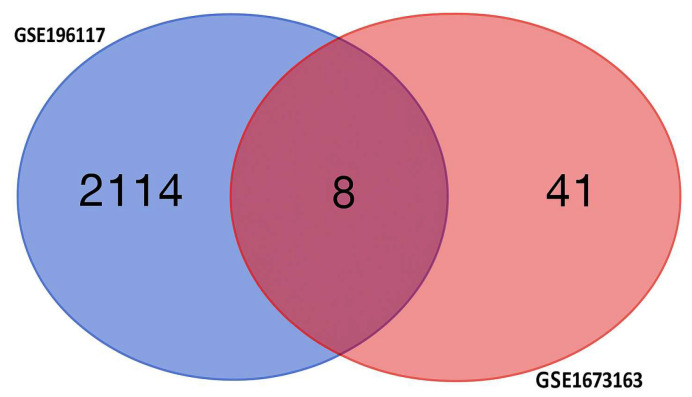
The Venn diagram shows differentially expressed genes (DEGs) specific to each condition, with non-overlapping numbers representing unique genes and the overlapping section highlighting genes that are expressed differentially across multiple condition.

Using two independent studies, RNA-Seq (GSE196117) and scRNA-seq (GSE167363), well-curated top expressed 2123 (Supplementary Table S1) and 50 (Supplementary Table S2) genes were obtained from the GSE19611 and GSE167363 studied, respectively. Among these genes, 2114 were expressed only in the RNA-Seq study group, while 41 were expressed only in the scRNA-Seq study, and eight genes were expressed in both studies, which shows in Figure 7. The eight differentially expressed genes (DEGs) as key diagnostic markers for bacterial sepsis: FTH1, B2M, NAMPT, KLF6, SRGN, S100A6, S100A9, and S100A8.

**Figure 8:**
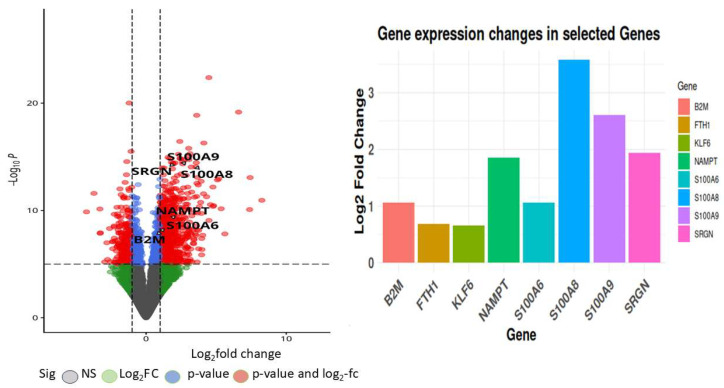
Volcano plot depicting the top commonly differentially expressed genes identified from the GSE196117 dataset, with log_2_ fold change plotted against −log_10_(*p*-value).

These differentially expressed genes (DEGs) exhibit unique functions across diverse immune cell types and disease outcomes, as indicated by their expression profiles and functional responsibilities. Figure 8 represent volcano plots of the genes that were differentially expressed in the two groups illustrate their distinct transcriptional profiles and nine-gene log fold value analysis revealed that up-regulated genes were involved in a sepsis. In healthy individuals, monocytes, T cells, and B cells demonstrate baseline immune surveillance, characterized by minimal ligand–receptor interactions. Genes like FTH1 regulate iron homeostasis to avert oxidative stress, B2M facilitates antigen processing and presentation, GAPDH propels glycolysis and metabolic processes, KLF6 modulates anti-inflammatory responses, and S100A6 enhances cellular stability and stress response. Dendritic cells in this cohort exhibit minimal immunological activation, with SRGN contributing to structural integrity and S100A8/S100A9 regulating baseline inflammation. In septic patients that survive, neutrophils and monocytes participate in augmented immune responses and tissue healing, supported by moderate ligand–receptor interactions. NAMPT facilitates energy generation via NAD+ synthesis, KLF6 regulates inflammation to aid recovery, and S100A8/S100A9 collaboratively enhance the immune response. In these individuals, NK cells and T cells demonstrate ligand–receptor interactions linked to immunological recovery, with GAPDH facilitating glycolysis, SRGN enhancing cell adhesion during tissue repair, and S100A6 supporting cellular recovery post-stress. In non-surviving septic patients, monocytes and macrophages exhibit hyperactivation of immunological responses, leading to cytokine storms and tissue destruction. Intense ligand–receptor interactions indicate significant immunological activation, with higher FTH1 levels to counteract oxidative stress, increased B2M for enhanced antigen presentation, and NAMPT promoting excessive metabolic demand, resulting in immune exhaustion. S100A8/S100A9 levels are significantly increased, exacerbating inflammation and cytokine signaling. Neutrophils and dendritic cells in this cohort demonstrate heightened ligand–receptor interactions, indicating immunological dysregulation. GAPDH facilitates metabolic requirements during stress, KLF6 is associated with tissue injury, SRGN promotes adhesion in inflamed tissues, and S100A6 acts as a biomarker for cellular stress in severe inflammatory states.

These genes showed consistent differential expression patterns across datasets, highlighting their potential as reliable biomarkers. Their involvement in immune modulation, inflammation, and oxidative stress pathways suggests a significant role in the pathophysiology of sepsis. This integrative analysis provides a robust foundation for further investigation into their diagnostic and prognostic value in bacterial sepsis.

**Figure 9:**
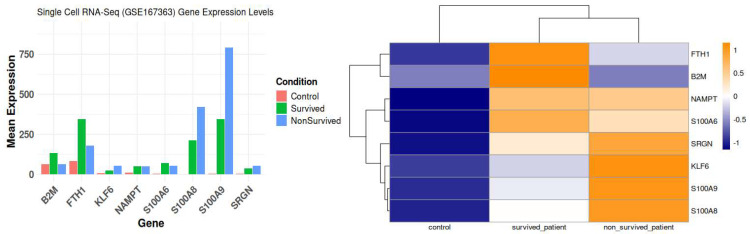
Condition specific eight commonly differentially expressed genes based on GSE167363; Heatmap of condition specific selected genes (scRNA).

We first investigated whether global transcriptional alterations were detectable between sepsis and control patients at different disease stages. As shown in Figure 9, this analysis identified S100A, a gene encoding the tissue-protective growth factor amphiregulin, as consistently among the top 50 differentially expressed genes (DEGs) across all sepsis patients compared to control samples.

The varied expression and functional importance of critical genes in sepsis, highlighting their ligand–receptor interactions across different patient groups: controls, sepsis survivors, and non-survivors.

The control group exhibited immunological function characterized by the standard interactions among monocytes, T cells, and B cells. Minimal ligand–receptor activity maintained homeostasis. Crucial functions such as iron homeostasis and antigen processing rely on genes such as FTH1 and B2M. S100A6 contributed to cellular stability and regulation of stress responses. At normal levels, S100A8 and S100A9 help control inflammation and keep iron levels steady in immune cells that are not performing anything. This illustrates their significance in maintaining immune system equilibrium under typical conditions.

In contrast, during the post-infection recovery phase, septic patients who lived exhibited significantly enhanced immune responses, especially in neutrophils and monocytes. In addition, the interactions between ligands and receptors were stronger, and genes like NAMPT and KLF6 were found to be very important in controlling the immune system and fixing damaged tissues. S100A8 and S100A9 exhibited a robust inflammatory response, with S100A9 collaborating with S100A8 to enhance immunological activity. This indicates that the immune system is vigilantly observing the circumstances, and tissues are recovering more rapidly as the sepsis diminishes.

The hyperactivation of the immune response in non-surviving septic patients resulted in a cytokine storm and considerable tissue damage. We observed increased ligand–receptor interactions, signifying enhanced immune activation and cytokine signaling. This was particularly evident in monocytes and macrophages, where the upregulation of genes including FTH1, B2M, and NAMPT signified an overstressed immune system. These patients showed markedly increased concentrations of S100A8 and S100A9, which instigated the inflammatory cascade and exacerbated the pronounced immunological dysregulation. Many interactions between ligands and receptors and overactivity of immune responses in neutrophils and dendritic cells are signs of a weak immune system and organ failure. Researchers have recognized S100A6 as a crucial indicator of cellular stress in these conditions.

The results show that S100A8 and S100A9 are important for the progression of sepsis, especially during the non-survivor phase, when they being strongly associated with inflammation and immune system dysregulation. Elevated levels in non-survivors indicate their potential as biomarkers for severe sepsis, reflecting a transition in the immune system from stimulation to fatigue, resulting in tissue and organ damage.

### 2.6. Drug Repurposing for the DEGs Related to Sepsis with PLAPT AI Tool

PLAPT python tool, a pre-trained model (https://github.com/Bindwell/PLAPT, accessed on 18 March 2025), has been used to calculate the negative log10 affinities between the proteins that corresponds to the nine DEGs founded in our study and 2958 ChEMBL approved drugs using a python script. The Uniprot IDs of proteins are P06702 (S100A9), P05109 (S100A8), P06703 (S100A6), P10124 (SRGN), P61769 (B2M), Q99612 (KLF6), P43490 (NAMPT), and P02794 (FTH1). The protein sequences, full list of approved drugs with SMILES formulas and other details, and the PLAPT affinities are presented as Supplementary Material “S1-PLAPT_affinities.xlsx”. Table 1 summarizes the 15 drugs identified as potential therapeutics, highlighting their interactions with all eight DEGs (proteins) associated with sepsis (negative log10 affinity > 8). The table also reports the mean negative log10 affinities, beginning with the drug showing the strongest interactions across all eight targets.

This AI predicts a drug directly linked to sepsis that could have a strong interaction with all eight DEGs—Itraconazole (an antifungal agent): fungal infections are a potentially involved in sepsis, and treating the underlying fungal infection is a direct approach in cases of fungal sepsis [21]. Additional prioritized drugs were mapped to sepsis-relevant mechanisms, including bile acid transporter inhibitors (Maralixibat, Maralixibat Chloride) with immunomodulatory effects [22], factor Xa anticoagulants (Apixaban, Edoxaban Tosylate) used to manage DIC in sepsis [23,24], antiviral protease inhibitors (Tipranavir, Darunavir Ethanolate) relevant for viral sepsis [25], a CGRP receptor antagonist (Atogepant) linked to vasodilatory and inflammatory pathways [26], a BCL-2 inhibitor (Venetoclax) reversing sepsis-associated immunosuppression [27], opioid-related agents (Naldemedine Tosylate, Levopropoxyphene Napsylate) with immune effects [28], drugs modulating vascular/cholinergic signaling (Dihydroergotamine, Scopolamine) [29], hormonal regulators such as Desogestrel [30] and Biotin (Vitamin B7), a metabolic cofactor with immunomodulatory roles [31,32]. Collectively, these results highlight drugs with established or emerging links to inflammation, coagulation, immune modulation, and infection control—key axes in sepsis pathophysiology.

- While additional drugs may not engage all proteins, they exhibit significant PLAPT affinity for particular targets relevant to sepsis.
- For S100A8 (Calgranulin A): Upadacitinib (neg_log10_affinity_M = 9.57) as JAK inhibitor—JAK-STAT signaling is a major pathway for cytokine production, and the “cytokine storm” is central to sepsis pathogenesis. Thus, this drug can directly modulate this inflammatory response [33]; Mometasone Furoate (neg_log10_affinity_M = 8.98) as a corticosteroid—corticosteroids have potent anti-inflammatory effects, and their use in sepsis is controversial, with potential benefits in some patients but risks in others [34].
- For S100A9 (Calgranulin B): Upadacitinib (neg_log10_affinity_M = 9.43) as a JAK inhibitor (see S100A8 explanation)—the PLAPT affinity is similar to the one for S100A8, predicting interaction with the complex of S100 protein family.
- For NAMPT (Nicotinamide phosphoribosyltransferase): Inosine (neg_log10_affinity_M = 10.45): Inosine is a purine nucleoside that can have immunomodulatory effects, often acting through adenosine receptors. It has shown some protective effects in preclinical models of sepsis, often by reducing inflammation and organ damage [35].
- For S100A6 (Calcyclin): Deucravacitinib (neg_log10_affinity_M = 10.80) as a TYK2 Inhibitor—it plays a role in immune and inflammatory responses [36]; Upadacitinib (neg_log10_affinity_M = 9.63)—See S100A8 explanation.
- For SRGN (Serglycin): Alpelisib (neg_log10_affinity_M = 10.78) as a PI3K inhibitor (primarily used in cancer treatment)—PI3K signaling is involved in immune cell activation and inflammation, and, even if this drug is not a sepsis treatment, its mechanism of action is relevant to inflammatory processes [37]; Inosine (neg_log10_affinity_M = 10.49)—see NAMPT explanation.
- For B2M (Beta-2-microglobulin): Inosine (neg_log10_affinity_M = 10.63)—see NAMPT explanation; Alpelisib (neg_log10_affinity_M = 9.99)—see SRGN explanation.

If we focus only on the S100A8 and S100A9 targets, PLAPT affinities suggested possible drug repurposing for other compounds in Table 2 (in addition to the interactions with all nine DEGs).

### 2.7. Molecular Docking and Molecular Dynamics Analysis

The best interactions predicted by the PLAPT AI model have been confirmed with molecular docking and molecular dynamics. Initial molecular docking analysis was performed to screen ligands targeting the S100-A9 protein (PDB ID: 4GGF), identifying promising drug candidates. The docking simulations revealed favorable binding free energies for both Eliquis (Apixaban) and Maralixibat chloride (Maralixibat), with Eliquis exhibiting a top binding affinity ranging from −8.6 to −6.8 kcal/mol and Maralixibat showing values from −8.1 to −5.8 kcal/mol (Table 3).

**Figure 10:**
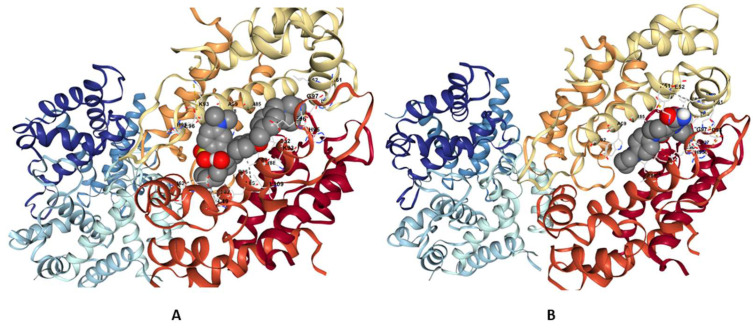
Three-dimensional interactions of 4GGF with Maralixibat chloride (Maralixibat) and Eliquis (Apixaban) (**A**,**B**).

Maralixibat chloride (Maralixibat) and Eliquis (Apixaban) demonstrated significant binding interactions with 4GGF by forming two conventional hydrogen bonds. These bonds were observed at bond lengths of 2.72 Å and 1.98 Å, involving the A chain residues of PHE48, LEU49, and ASP65, respectively. In Additionally, Eliquis intrect with HIS27, ASN47, PHE48. The docking position of ligands are presented in Figure 10.

**Figure 11:**
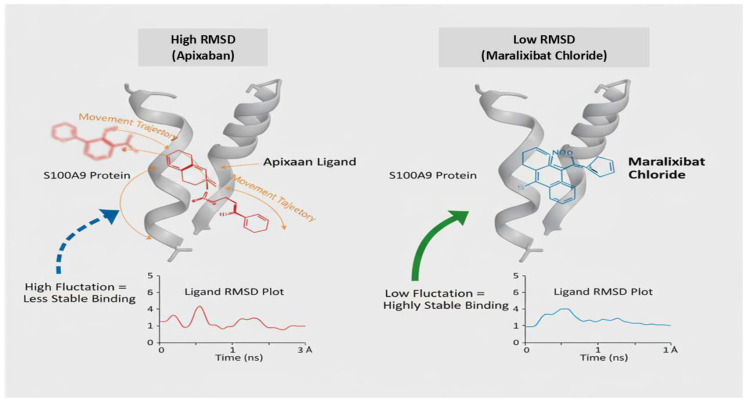
Stability Profiles of S100A9 Complexes: Apixaban versus Maralixibat Chloride (200 ns MD Simulation).

Based on these strong binding affinities, Eliquis and Maralixibat were selected as top candidates for further detailed analysis. The Molecular Dynamics (MD) simulations, performed over a robust 200 nanoseconds (ns), clearly demonstrate a hierarchy of binding stability between the S100A9 protein (4GGF) and the two test ligands, Apixaban and Maralixibat Chloride. Analysis of the Ligand Root Mean Square Deviation (RMSD) revealed that the Apixaban complex maintained a fluctuating RMSD between 2.0 A° and 3.0 A° after initial equilibration. This indicates that while the ligand remains bound, it undergoes noticeable conformational motion and explores a larger volume within the pocket. In stark contrast, the Maralixibat Chloride complex displayed exceptional long-term rigidity, with its ligand RMSD consistently confined to a tight, low-fluctuation range of 1.1 A° to 1.5 A° over the entire 200 ns trajectory. This minimal deviation confirms a highly specific, deep, and energetically favorable binding pose (see Figure 11).

This enhanced stability was corroborated by the protein’s internal dynamics. The global Protein RMSD for the Maralixibat Chloride complex remained consistently below 1.5 A°, suggesting a more stable backbone compared to the Apixaban complex (which fluctuated up to 2.5 A°). Furthermore, the Root Mean Square Fluctuations (RMSF) confirmed that Maralixibat Chloride imparts superior structural rigidity to the protein: most residues in this complex fluctuated below 1.0 A°, whereas the Apixaban complex showed moderate flexibility with average fluctuations around 1.0 A° to 2.0 A° (see Figure 11). Collectively, these results establish Maralixibat Chloride as forming a significantly more stable and rigid complex with S100A9. These results are summarized in Supplementary Figures S7–S16, which illustrate the RMSD, RMSF, Rg, hydrogen bond profile, and potential energy analyses of the S100A9–Eliquis complex over the simulation trajectory.

## 3. Discussion

### 3.1. NFκB1 as a Regulatory Transcription Factor and Its Target Genes in Sepsis Pathophysiology

Members of the S100 protein family, S100A8 [10,11,38], S100A9 [39,40,41] and S100A6 [42] are essential to the pathophysiology of sepsis. These calcium-binding proteins are important damage-associated molecular patterns (DAMPs) [43,44] that trigger inflammatory reactions. They frequently form a heterodimer called calprotectin, and their expression is noticeably increased in sepsis, leading to tissue destruction and immunological dysregulation. S100A8/A9 proteins activate downstream signaling cascades, including NF-κB and MAPK, by binding to receptors like Toll-like receptor 4 (TLR4) [45,46] and the receptor for advanced glycation end products (RAGE) [47]. Pro-inflammatory cytokines like IL-6, TNF-α, and IL-1β are released as a result of this activation, intensifying the inflammatory cascade. Additionally, these proteins encourage the migration of neutrophils and monocytes to infection sites, which is a defining feature of the hyper-inflammatory response in sepsis [48].

NFκB1 and KLF6 synergistically amplify the inflammatory response in sepsis. NFκB1 is a master regulator of cytokine production, while KLF6 enhances NFκB-driven transcription of inflammatory genes, leading to an excessive immune response. S100A8/A9-mediated NFκB activation further upregulates KLF6, creating a positive feedback loop that exacerbates inflammation. Additionally, KLF6 itself can induce NFκB1 nuclear translocation, amplifying the production of TNF-α and IL-6.

### 3.2. The Roles of S100A8 and S100A9 in the Development of Sepsis Complemented by Other Genes

Another member of the S100 family, S100A6, contributes to the cytokine storm that characterizes sepsis by being linked to stress and inflammatory reactions. Similarly, by preserving iron homeostasis, which is essential for reducing the tissue-damaging effects of reactive oxygen species (ROS), ferritin heavy chain (FTH1) aids in the regulation of oxidative stress.

During inflammation, NAMPT (nicotinamide phosphoribosyltransferase [49,50] a crucial regulator of NAD+ production [51], supports the energy-intensive mechanisms underlying cytokine synthesis and immune cell activity, which are significantly elevated in sepsis. Serglycin (SRGN) promotes inflammation and immune cell activity by facilitating the release and storage of inflammatory mediators [52,53].

Beta-2-microglobulin (B2M) supports immune surveillance by aiding in antigen presentation [54], while GAPDH, despite being primarily a glycolytic enzyme, also contributes to inflammation by regulating cytokine production during metabolic stress [55]. TMSB10 [56] and VIM [49] as cytoskeletal regulators, they support immune cell migration and tissue remodeling, which are critical for immunological defense and sepsis pathogenesis.

These pathways’ convergence emphasizes how crucial S100A8 and S100A9 are in coordinating the inflammatory response during sepsis. Excessive inflammation, tissue damage, and multi-organ failure result from their overexpression and interactions with other important mediators, which intensify the immune activation feedback loop. Improving clinical outcomes and reducing the hyper-inflammatory response in sepsis can be achieved by targeting S100A8/A9 [57] and the pathways that are linked to it. Together with other genes like FTH1, NAMPT, and SRGN, these proteins are also useful indicators for treatment monitoring and early identification in septic patients.

### 3.3. AI-Guided Drug Repurposing Targeting S100A8/A9 in Sepsis

- Upadacitinib, a Janus kinase (JAK) inhibitor, represents a newer class of drugs that target cytokine signaling, a major driver of the “cytokine storm” characteristic of severe sepsis. Opioid-related drugs, exemplified by the opioid antagonist Naldemedine, point to the complex interplay between pain management, opioid use, and immune function in critically ill patients. Bile acid modulators, such as Maralixibat, suggest a potential role for bile acid signaling in modulating inflammation, whereas CGRP antagonists like Atogepant indicate a potential indirect mechanism by which CGRP modulation could influence sepsis outcomes. Additionally, biotin has been implicated in S100A8/A9 biology, as these proteins are biotinylated and secreted by human neutrophils. Biotin deficiency may impair immune responses by reducing cytokine production, including IL-2 and IFN-γ, by T-cells.
- Among these candidates, the JAK inhibitor Upadacitinib stands out, with predicted −log10 affinities of 9.57 and 9.43 for S100A8 and S100A9, respectively. This highlights its potential as a promising therapeutic option for sepsis, given the established role of JAK-STAT signaling in cytokine storm pathogenesis.
- To assess the predictive accuracy of PLAPT AI, we compared its predictions with experimentally determined S100A9 affinities for compounds currently under investigation but not yet approved. Tasquinimod (CHEMBL2107784), in clinical trial phase 3, binds S100A9 with a dissociation constant (Kd) of 30–70 nM, as measured by surface plasmon resonance, thereby disrupting interactions with RAGE and TLR4 and modulating inflammatory responses [58]. The experimentally transformed –log10 values (7.52–7.15 M) exceed PLAPT’s prediction of 6.26 M. Similarly, Paquinimod (CHEMBL67776), in clinical trial phase 2, shows an IC50 of approximately 0.5 µM in a TR-FRET assay by inhibiting S100A9 interactions with TLR4 and RAGE, reducing leukocyte infiltration and inflammation [59]. The transformed –log10 value of 6.3 M is higher than PLAPT’s predicted 5.11 M, indicating that the AI-based predictions, while informative, may underestimate true binding affinities. These findings illustrate that, although discrepancies exist between experimental measurements and PLAPT AI predictions, the model consistently captured strong molecular interactions relevant to sepsis. For example, the predicted –log10 affinity for Paquinimod–S100A9 (4–6 M) falls within the initial screening range, while Tasquinimod–S100A9 (8–10 M) aligns with the lead optimization stage. This stratification suggests that AI-driven forecasts can not only approximate experimental binding but also help prioritize compounds at different phases of the drug development pipeline.
- The predicted binding of several approved drugs to S100A8 and S100A9 further underscores the potential for drug repurposing in sepsis. Yet, the scope of these results is constrained by the limitations of the AI model itself. Thus, future work should integrate computational approaches, such as in vivo studies, to confirm these interactions and evaluate their therapeutic relevance.
- Collectively, these observations highlight promising candidates for S100A8/A9-targeted repurposing in sepsis. Although the extracellular localization of these proteins presents inherent challenges for direct small-molecule inhibition, our AI-based predictions provide a rational framework for advancing antibody-based, peptide-based, or targeted-delivery therapeutic strategies. By bridging AI-driven insights with experimental validation, this approach holds the potential to accelerate the identification of effective interventions for one of the most pressing unmet needs in critical care medicine.

### 3.4. Therapeutic Potential for Sepsis-Induced Immunomodulation

- Sepsis represents a multifaceted inflammatory disorder characterized by immune dysregulation, endothelial dysfunction, and metabolic imbalance. Effective therapeutic strategies should therefore target both inflammatory and metabolic axes to restore immune homeostasis. In this context, Maralixibat chloride and Apixaban (Eliquis) demonstrate promising but distinct immunomodulatory potentials, with Maralixibat chloride emerging as the more potent and stable candidate based on our computational analyses.
- Maralixibat chloride, a selective inhibitor of the ileal bile acid transporter (IBAT; SLC10A2), modulates systemic bile acid metabolism—an increasingly recognized regulator of immune signaling [60]. By reducing bile acid reabsorption in the ileum, Maralixibat alters gut–liver communication and dampens key inflammatory pathways, including TLR4–NF-κB activation and NLRP3 inflammasome signaling. This modulation can alleviate sepsis-associated intestinal barrier dysfunction, endotoxemia, and hepatic inflammation. Notably, molecular docking and long-term molecular dynamics (MD) simulations demonstrated that Maralixibat binds stably to the S100A9 protein, a critical DAMP driving sepsis-associated immune suppression. The Maralixibat–S100A9 complex exhibited minimal ligand RMSD, reduced protein flexibility (RMSF), and high structural rigidity, indicating exceptional conformational stability. This strong and sustained binding suggests that Maralixibat effectively locks S100A9 in an inactive state, thereby blocking its pro-inflammatory signaling through RAGE and TLR4 pathways. Such stable inhibition may prevent the progression from hyperinflammation to immunosuppression, offering a durable therapeutic effect [61].
- Apixaban (Eliquis), a clinically approved Factor Xa inhibitor, also displayed favorable binding interactions with S100A8/A9 in silico, reflecting its potential anti-inflammatory and endothelial-protective effects in sepsis [24,62]. The S100A8/A9–Apixaban complex remained dynamically stable during 200 ns simulations, suggesting partial inhibition of pro-inflammatory signaling. However, compared to Maralixibat, the Apixaban complex showed higher ligand fluctuation and lower overall stability, indicating a more transient binding interaction. While Apixaban’s dual anticoagulant and anti-inflammatory profile is therapeutically valuable, its weaker persistence within the S100A9 binding pocket suggests that its immunomodulatory effect may be secondary or supportive rather than central in sepsis management [63]. Overall, the comparative analysis highlights Maralixibat chloride as the more impactful immunomodulatory candidate, capable of exerting long-term stabilization and inhibition of S100A9 activity. Its ability to integrate metabolic regulation with immune suppression control positions it as a novel dual-acting therapeutic agent for sepsis. Although computational results provide strong mechanistic insights, experimental validation using surface plasmon resonance (SPR), isothermal titration calorimetry (ITC), and cytokine-based cell assays will be critical to confirm these interactions and establish Maralixibat’s translational potential for sepsis-induced immunomodulation.

### 3.5. Limitations of the Study and Directions for Future Research

This study presents several important limitations. Our analyses are exploratory and primarily hypothesis-generating, relying on computational predictions rather than direct mechanistic validation. While the AI-driven drug repurposing framework and transcriptomic analyses provide new insights into S100A8/A9 biology in sepsis, they may not fully capture the complexity of in vivo immune responses. The relatively small sample sizes of the RNA-seq and scRNA-seq datasets also limit statistical power and generalizability, underscoring the need for validation in larger, independent patient cohorts with well-defined clinical characteristics. Furthermore, the absence of functional assays prevents direct correlation of predicted drug–target interactions with immune modulation or therapeutic efficacy. Experimental validation through in vitro binding, immune functional assays and in vivo models will therefore be essential to substantiate these findings. In future work, the integration framework can be expanded by incorporating cell-type-resolved pseudo-bulk datasets, CIBERSORTx deconvolution [64], and trajectory-based analyses to better resolve the temporal and cellular dynamics of the sepsis response. Such approaches, combined with larger, harmonized cohorts and standardized clinical metadata, will enhance the biological resolution and translational value of multi-omic sepsis studies

Notwithstanding these limitations, the study provides a framework for integrating AI-guided predictions, molecular docking and MD with transcriptomic profiling to prioritize therapeutic candidates. Addressing the gaps in sample size, mechanistic understanding, and functional validation will enhance the translational potential of our results and accelerate the development of clinically actionable interventions in sepsis

## 4. Materials and Methods

### 4.1. Data Collection

We combined scRNA-seq GSE167363 [65] and bulk RNA-seq GSE196117 [66] datasets to elucidate complementary facets of sepsis biology. The datasets were obtained from the NCBI Gene Expression Omnibus (GEO) repository [67]. These datasets were selected for integrative analysis based on their relevance to whole-blood gene expression in septic patients (survivors and non-survivors) and healthy controls. Both datasets were harmonized for key clinical metadata, including disease status and survival outcome, while variables such as male and comorbidities were excluded due to incomplete annotation. The scRNA-seq dataset comprised peripheral blood mononuclear cells (PBMCs) from 10 patients with Gram-negative sepsis (including both survivors and non-survivors) and 2 healthy controls, enabling high-resolution characterization of cell-type-specific transcriptional dynamics. In parallel, the bulk RNA-seq dataset consisted of whole blood samples from 33 patients with sepsis and 7 healthy controls, providing a systems-level perspective on transcriptional changes during therapeutic intervention.

**Figure 12:**
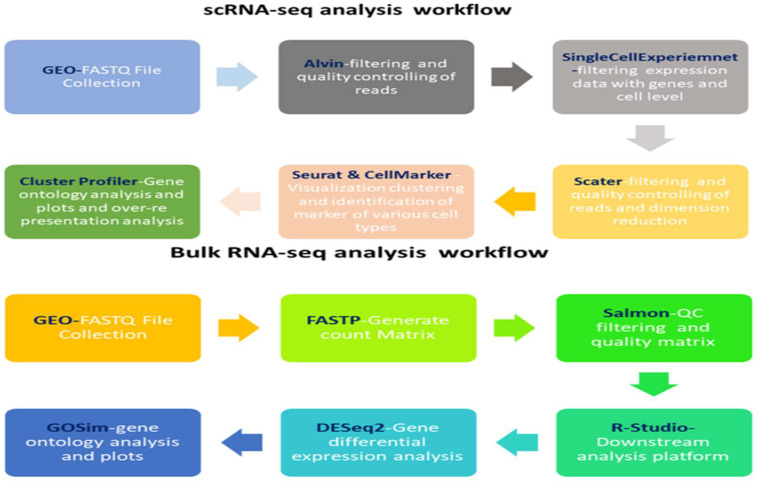
scRNA-seq and RNA-seq workflow: The workflow emphasizes scRNA-seq’s ability to resolve cellular heterogeneity versus RNA-seq’s capacity for system-wide transcriptional insights.

Because both datasets profiled circulating leukocytes, we used the scRNA-seq data to derive robust cell-type marker signatures, which were then applied to (i) deconvolve bulk expression profiles, (ii) construct pseudo-bulk summaries for cross-platform comparison, and (iii) evaluate whether bulk-level transcriptional alterations reflected shifts in cellular composition or within-cell-type reprogramming. Comprehensive clinical metadata are available in the original publications, with cohort characteristics summarized and accession details provided in Table 4. A schematic overview of the integrative analysis workflow is presented in Figure 12.

### 4.2. scRNA-Seq Data Processing and Analysis

Raw single-cell RNA-seq data from GSE167363 were processed using the Salmon suite [68] for transcript quantification. Downstream analyses were conducted in R (v4.X) using Seurat Version 4.1 [69], following a structured workflow to ensure reproducibility and methodological clarity. Quality control retained cells expressing >750 genes, >1000 transcripts, and <5% mitochondrial RNA content. Normalization and feature selection were performed with NormalizeData and ScaleData, and the top 2000 highly variable genes were identified using FindVariableFeatures [70]. Batch effects were corrected with Harmony [71], and dimensionality reduction was achieved via principal component analysis (PCA) followed by UMAP for clustering and visualization.

Cell-type identification was based on cluster-specific marker genes detected with FindAllMarkers (log2FC > 0.25, min.pct > 0.25). The top 50 markers per cluster were annotated using CellMarker 2.0 [72] and validated against canonical immune cell markers. Differential expression analyses between sepsis and control samples were conducted using FindMarkers (Wilcoxon test; *p* < 0.05, |log2FC| > 0.5). To explore intercellular communication, the CellChat package Version 1.5.0, [73] was employed, with network visualization implemented through patchwork Version 1.3.2 [74].

### 4.3. Bulk RNA-Seq Data Processing and Analysis

An analytical pipeline was uniformly applied to the bulk RNA-seq dataset GSE196117. The assessment of raw reads was conducted with FastQC v0.11.9 (http://www.bioinformatics.babraham.ac.uk/projects/fastqc, accessed on 18 March 2025), focusing on evaluating per-base sequence quality, GC content, sequence length distribution, and the presence of adapter contamination. Reads underwent trimming and filtering using fastp v0.23.0, applying a Phred score threshold of ≥15 and a minimum transcript length of 20 bp. Transcript-level abundances were quantified utilizing Salmon v1.10.0, and gene-level counts were compiled for subsequent analyses. Normalization across samples was executed utilizing the trimmed mean of M-values (TMM) to address variations in library size [75], and TPM values were computed for visualization and relative expression comparisons. The τ metric was employed to assess tissue specificity for each gene. Downstream analyses and visualizations, such as heatmaps, were performed in R Studio v4.1 utilizing the GSVA package [76,77]. An analysis of differential gene expression (DEG) was performed comparing 33 sepsis patients to seven healthy controls, utilizing DESeq2 v1.36.0 [78]. The thresholds applied were |log2FC| > 0.5 and an adjusted *p*-value < 0.05. Volcano plots were created utilizing ggplot2 version 3.5.0. Functional enrichment analyses, such as Gene Ontology (GO) [79], KEGG [80,81] and pathway over-representation analysis (ORA), were conducted utilizing clusterProfiler v4.2.2 [82], incorporating gene sets from the MSigDB Hallmark collection (v7.4) [83]. The full ORA results can be found in Supplementary Table S3. Differentially expressed genes were evaluated in relation to the overall gene universe, with adjusted *p*-values provided to account for multiple testing corrections [84]. All analyses were conducted and interpreted using R Studio.

### 4.4. Artificial Intelligence Prediction of Approved Drugs—Sepsis Protein Interactions

To explore potential interactions of existing drugs for repurposing with the best-predicted proteins, an AI-based tool titled Protein–Ligand Binding Affinity Prediction Using Pretrained Transformers (PLAPT) [85] was utilized to predict binding affinities (expressed as negative log10 affinity). PLAPT predicts the binding affinity of ligand–protein complexes using SMILES formulas for ligands and FASTA amino acid sequences for proteins. The model integrates two pre-trained transformers: ProtBERT for protein sequences and ChemBERTa for SMILES formulas of drugs. These transformers convert both inputs into embeddings for a regressor model, pre-trained to predict interaction affinities.

Using the PLAPT Python package, (https://github.com/Bindwell/PLAPT, accessed on 18 March 2025) drug–protein affinities were calculated for 2958 ChEMBL [86] approved drugs with molecular masses between 200 and 900. These drugs were screened for potential repurposing against all proteins linked to sepsis identified from the integrated transcriptomic analysis in this study. FASTA protein sequences corresponding to genes linked to sepsis were downloaded using the API interface of the UniProt database [87]. A cutoff of 8 for the negative log10 affinity was used to aim for potent, selective compounds with drug-like properties [88]. To allow direct comparison with the neg_log10_affinity_M values from your work, our values and other affinities from previous works needed to be on the same scale. Thus, a −log10 transformation was applied to the reported literature affinities (Kd, IC50, EC50), resulting in their negative log10 values (pKd, pIC50, pEC50).

To contextualize PLAPT-AI affinity predictions, we adopted reference thresholds commonly applied in drug discovery. In early initial screening, affinities of 4–6 (−log10 M; 100 µM–1 µM) typically capture weak but detectable binders. Fragment screening relies on very low-affinity interactions (3–5; 1000 µM–10 µM) combined with structural data to enable optimization. The hit-to-lead stage aims for moderate binding improvements (6–8; 1 µM–10 nM), while lead optimization progresses toward high-affinity, drug-like compounds (8–10+; 10 nM–0.1 nM or lower). Finally, clinical candidates generally exhibit sub-nanomolar potency (≥9; <1 nM), where binding strength is balanced with absorption, distribution, metabolism, excretion, and safety (ADME/Tox) properties. These thresholds were used as interpretive benchmarks for evaluating AI-predicted interactions with S100A8/A9.

### 4.5. Molecular Docking and Molecular Dynamics Simulations

Molecular docking was performed using AutoDock Vina version 1.2.4 [89] to evaluate interactions between Protein S100-A9 (PDB ID: 4GGF) [90] and the ligand structure of Apixaban (D03213) and Maralixibat (DB16226) was retrieved from DrugBank. The energy-minimized prior to docking the ligands Eliquis (Apixaban) and Maralixibat chloride (Maralixibat). Proteins were preprocessed by removing water molecules, adding polar hydrogens, and assigning Gasteiger charges, while ligands were energy-minimized and converted to PDBQT format. Docking grids were centered on the active sites and co-crystallized ligand positions to encompass the binding pockets fully. Docked poses were ranked by binding affinity, and the two top-scoring complexes were selected for further molecular dynamics (MD) simulations.

MD simulations were conducted using GROMACS version 2025.3 with the CHARMM36 version c23f2 [91] force field, following established protocols [92]. Protein–ligand complexes were prepared and visualized in PyMOL, v 3.1. [93] and system parameters were generated for compatibility with GROMACS. Comparative analyses of the unbound proteins and their complexes were performed to assess structural stability, conformational dynamics, and ligand-induced effects throughout the simulation period.

## 5. Conclusions

Integrating NFκB1 with its regulatory targets provides a comprehensive framework to understand the multifaceted pathophysiology of sepsis, including immunological dysregulation, oxidative stress, metabolic imbalance, and tissue remodeling. Our analyses highlight key genes—FTH1, B2M, NAMPT, KLF6, SRGN, S100A6, S100A8, and S100A9—as central modulators of these processes. FTH1 mitigates oxidative stress by limiting free iron accumulation, while B2M, a component of MHC-I, is essential for immune surveillance but can exacerbate inflammation when overexpressed. NAMPT, a critical NAD+ biosynthetic enzyme, may drive cytokine storm via NFκB signaling. KLF6 contributes to endothelial dysfunction and tissue repair, and SRGN amplifies inflammatory responses through cytokine release. S100 proteins, including S100A6 and the calprotectin complex (S100A8/A9), regulate immune cell recruitment and ROS production, acting through the TLR4/NFκB pathway and serving as potential biomarkers of disease severity.

Collectively, these genes and pathways reveal the intricate crosstalk between oxidative stress, metabolic regulation, and inflammation in sepsis. While protective mechanisms such as FTH1 and KLF6 may mitigate tissue damage, targeting the NFκB1–B2M–NAMPT–S100A6 axis and associated pathways like TLR4/NFκB represents a promising strategy to modulate hyperinflammation. Pathway enrichment analyses further underscore the relevance of these targets for both therapeutic intervention and biomarker development.

Leveraging these insights, we applied an AI-based drug repurposing approach using PLAPT to evaluate interactions between 8 key protein targets and 2958 ChEMBL-approved compounds. The best interactions have been verified with molecular docking and MD. The analysis identified strong predicted interactions with functional relevance to sepsis, suggesting opportunities to repurpose existing drugs to modulate critical pathways. This integrative transcriptomic and computational framework provides a hypothesis-generating foundation for developing targeted, clinically actionable interventions aimed at improving outcomes in sepsis patients.

## Figures and Tables

**Table 1:** PLAPT AI negative log10 affinities of the approved drugs that interacts with all eight DEGs (ordered by the mean negative log10 affinity).

Drug ID	Drug Name	FTH1	S100A8	S100A9	S100A6	SRGN	B2M	KLF6	NAMPT	Mean
CHEMBL17879	Maralixibat Chloride	10.67	10.72	11.17	10.45	10.57	11.30	11.15	11.42	10.99
CHEMBL363392	Maralixibat	10.00	9.93	10.11	10.02	9.45	10.21	10.09	10.39	10.05
CHEMBL231779	Apixaban	9.78	10.44	10.71	9.09	9.72	10.16	10.71	10.35	10.29
CHEMBL222559	Tipranavir	10.40	10.36	10.32	10.35	9.34	9.83	10.31	9.92	10.13
CHEMBL2105682	Edoxaban Tosylate	9.75	9.50	9.31	9.77	8.59	8.54	9.32	8.51	9.15
CHEMBL64391	Itraconazole	9.67	9.79	9.95	9.68	9.26	9.21	9.94	9.26	9.65
CHEMBL3991065	Atogepant	9.31	9.55	9.58	9.06	8.25	8.76	9.58	9.02	9.18
CHEMBL857	Biotin	10.37	10.32	10.23	10.37	8.55	8.88	10.23	9.20	9.81
CHEMBL3039508	Naldemedine Tosylate	10.67	10.08	9.46	10.99	8.24	8.34	9.50	8.38	9.40
CHEMBL3137309	Venetoclax	9.44	9.55	9.52	9.23	8.38	8.45	9.54	8.43	9.08
CHEMBL1593906	Levopropoxyphene Napsylate	10.07	9.72	9.38	10.15	8.66	8.27	9.40	8.42	9.24
CHEMBL1200517	Dihydroergotamine Mesylate	10.04	9.65	9.27	10.18	8.14	8.23	9.30	8.26	9.10
CHEMBL2251240	Scopolamine Hydrobromide	9.71	9.59	9.25	9.71	8.61	8.35	9.27	8.90	9.14
CHEMBL1201127	Darunavir Ethanolate	10.43	9.71	8.93	10.71	8.66	8.21	8.97	8.23	9.17
CHEMBL1533	Desogestrel	9.11	8.96	8.90	9.04	8.45	8.27	8.91	8.65	8.79

**Table 2:** PLAPT AI negative log10 affinities of the approved drugs that interacts with S100A8 and S100A9 (ordered by the mean negative log10 affinity).

Drug ID	Drug Name	Official Drug Medical Indication	Link to Sepsis/Inflammation	S100A8	S100A9	Mean
CHEMBL46286	Omacetaxine Mepesuccinate	Treatment of chronic myeloid leukemia (CML) resistant to tyrosine kinase inhibitors	Indirect: Protein synthesis inhibitor. While not a direct sepsis treatment, it highlights the potential role of protein synthesis modulation in inflammatory conditions.	9.89	9.47	9.68
CHEMBL1580	Pentostatin	Treatment of hairy cell leukemia	Indirect: Adenosine deaminase inhibitor, leading to accumulation of deoxyadenosine, which is toxic to lymphocytes. Can have immunosuppressive effects.	9.64	9.59	9.62
CHEMBL3989716	Propoxyphene Napsylate	Opioid Analgesic	Indirect: Opioids have immunomodulatory effects.	9.66	9.39	9.53
CHEMBL5315119	Upadacitinib Hemihydrate	Treatment of rheumatoid arthritis, psoriatic arthritis, atopic dermatitis, ulcerative colitis, Crohn’s disease	Direct: JAK inhibitor, modulating cytokine signaling. Cytokine storm is a key feature of sepsis.	9.57	9.43	9.50
CHEMBL3039514	Elbasvir	Treatment of hepatitis C virus (HCV) infection (NS5A inhibitor)	Indirect: Antiviral. Viral infections can trigger sepsis. Elbasvir inhibits Hepatitis C Virus (HCV) NS5A protein. While not directly used for sepsis, chronic HCV infection can have systemic inflammatory effects.	9.48	9.37	9.43
CHEMBL2107004	Quinestradiol	Estrogen replacement therapy	Indirect: Estrogen has immunomodulatory effects, and its levels can influence inflammatory responses (mixed results).	9.19	9.18	9.19

**Table 3:** Top hit interactions of 4GGF with ligands.

PDB ID	Compound Name	Binding Affinity (kcal/mol)	Number of H-Bonds/Key Residues
4GGF	Maralixibat chloride (Maralixibat)	−9	PHE48, LEU49, GLU52, VAL58, HIS61, ILE62, ASP65, LEU82, ARG85
4GGF	Eliquis (Apixaban)	−8	HIS27, ASN47, PHE48, LEU49, LYS50, LYS51, GLU52, ILE62

**Table 4:** Samples used in the scRNA-seq analysis.

Type	Study	Sepsis Samples	Control Samples
Bluk RNA Seq	GSE196117	33 (GSM5860293, GSM5860292, GSM5860291, GSM5860290, GSM5860289, GSM5860288, GSM5860287, GSM5860286, GSM5860285, GSM5860284, GSM5860283, GSM5860282, GSM5860281, GSM5860280, GSM5860279, GSM5860278, GSM5860277, GSM5860276, GSM5860275, GSM5860274, GSM5860306, GSM5860305, GSM5860304, GSM5860303, GSM5860302, GSM5860301, GSM5860300, GSM5860299, GSM5860298, GSM5860297, GSM5860296, GSM5860295, GSM5860294)	7 (GSM5860273, GSM5860272, GSM5860271, GSM5860270, GSM5860269, GSM5860268, GSM5860267)
scRNA seq	GSE167363	10 (GSM5102902, GSM5102903, GSM5102904, GSM5102905, GSM5511351, GSM5511352, GSM5511353, GSM5511354, GSM5511355, GSM5511356)	2 (GSM5102900, GSM5102901)

## Data Availability

The data presented in this study are available in the NCBI Gene Expression Omnibus (GEO) at https://www.ncbi.nlm.nih.gov/geo/ (accessed on 3 March 2025), under accession numbers GSE196117 (bulk RNA-seq) and GSE167363 (single-cell RNA-seq). These datasets were derived from publicly available resources [GEO] [https://www.ncbi.nlm.nih.gov/geo/query/acc.cgi?acc=%20GSE196117 (accessed on 3 March 2025), https://www.ncbi.nlm.nih.gov/geo/query/acc.cgi?acc=GSE167363 (accessed on 3 March 2025)] [GSE196117, GSE167363].

## Supplementary Materials

The following supporting information can be downloaded at: https://www.mdpi.com/article/10.3390/ijms262211186/s1.

## Abbreviations

The following abbreviations are used in this manuscript:

S100A8/A9-S100 protein family contains calcium-binding proteins	
Sepsis-induced organ injury	
TNF-NF-κB	Tumor Necrosis Factor (TNF) and Nuclear Factor-kappa B (NF-κB)
GO	Gene Ontology
DEG	Differentially Expressed Genes
TLR4	Toll-Like Receptor 4
RAGE	Receptor for Advanced Glycation End Products
TNFAIP3	Tumor Necrosis Factor Alpha-Induced Protein 3
DAMPs	Damage-Associated Molecular Patterns
