# Supplementary material for: Integrated Transcriptomic Analysis of S100A8/A9 as a Key Biomarker and Therapeutic Target in Sepsis Pathogenesis and AI Drug Repurposing
Source: Int J Mol Sci. 2025 Nov 19;26(22):11186. doi: 10.3390/ijms262211186 (PMC12653820; doi:10.3390/ijms262211186)
Supplement: Supplementary file 1 [file ijms-26-11186-s001.zip › Supplementary Figures S1-S6.pdf]

Control

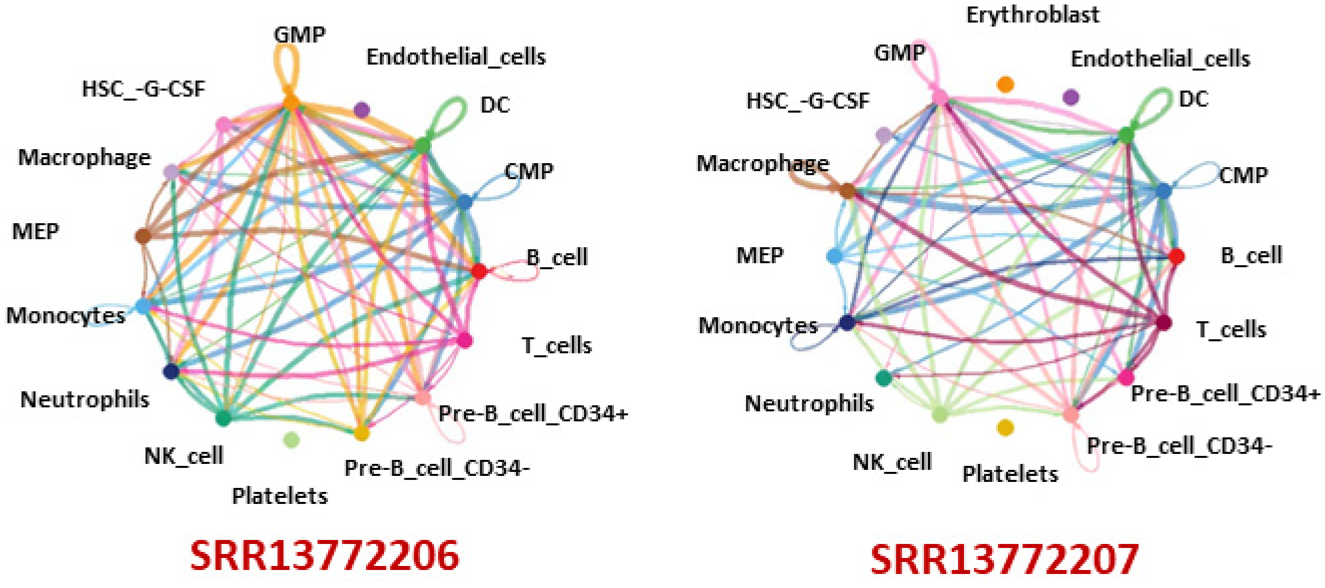

Supplementary Figure S1. Cell-cell communication network representation for Control Group showing normal interactions between immune cells, including T cells, macrophages, and endothelial cells, with balanced signaling pathways.

Survived_Septic_Patient

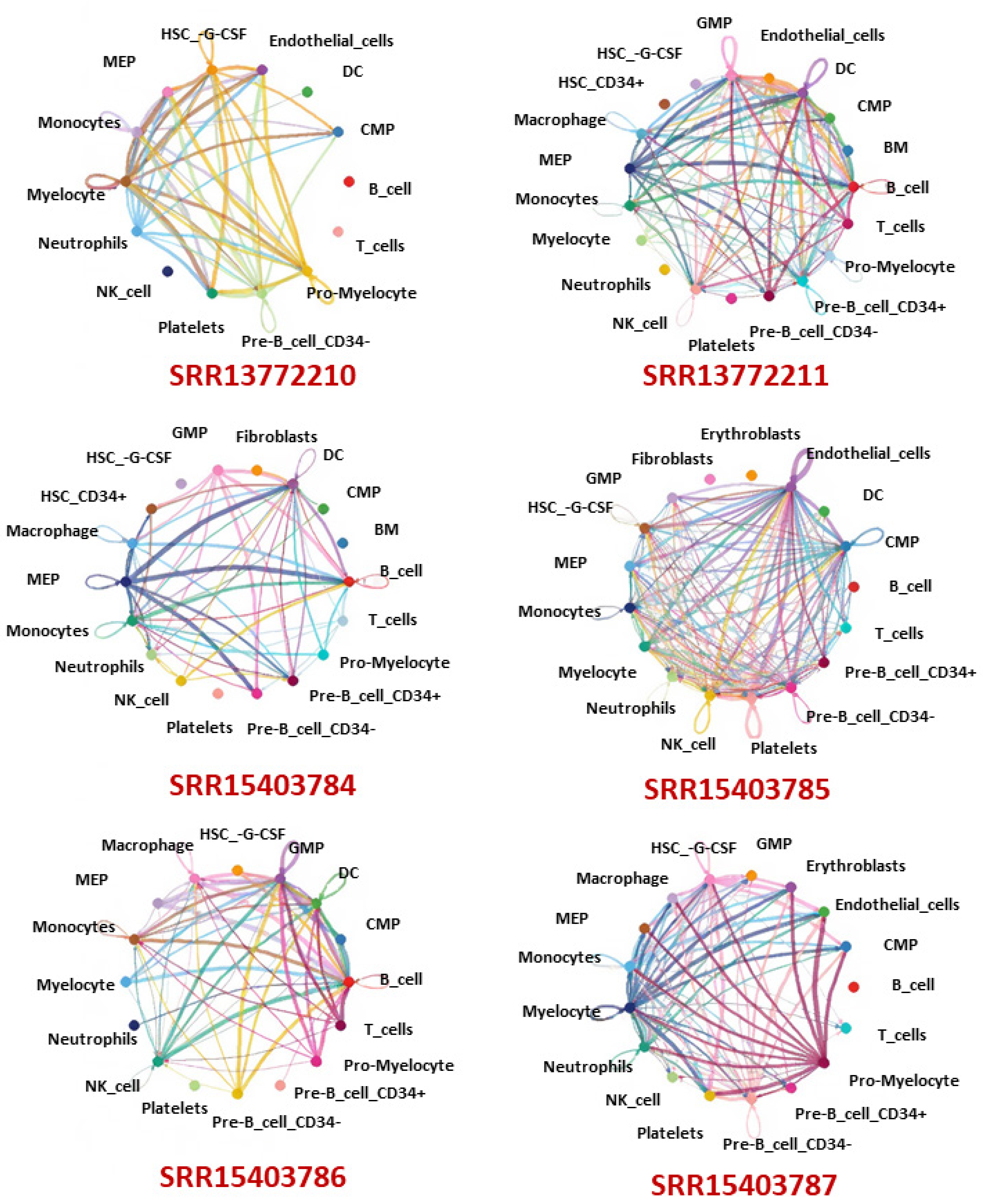

Supplementary Figure S2. Cell-cell communication network representation for Survived Septic Patients shows Increased immune activation and altered communication patterns, with heightened interactions among macrophages, neutrophils, and inflammatory pathways, suggesting immune adaptation post-sepsis.

Non_Survived_Septic_Patient

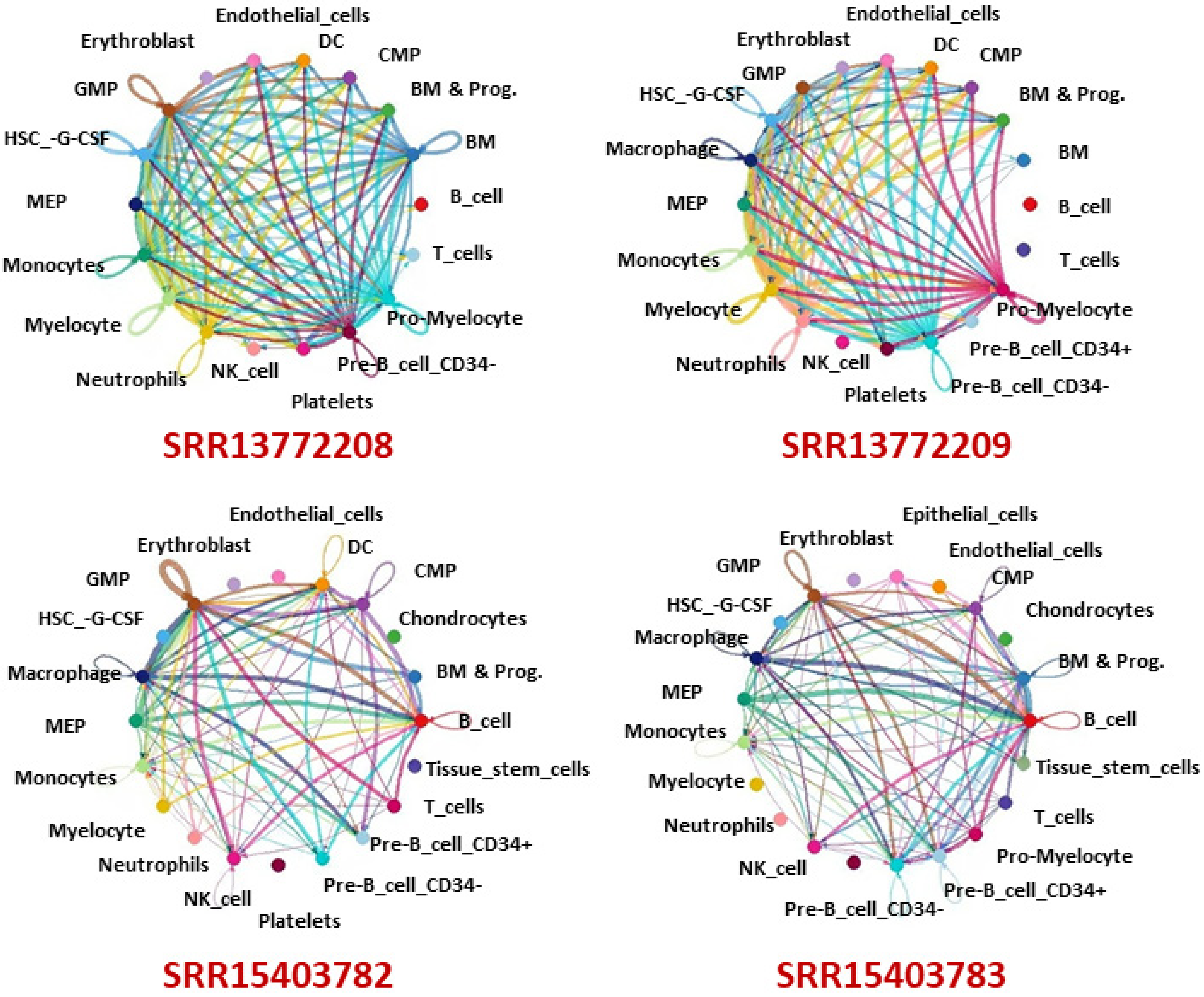

Supplementary Figure S3. Cell-cell communication network representation for Non-Survived Septic Patient group shows hyperactive neutrophil and monocyte signaling, disrupted endothelial interactions, and excessive inflammatory responses, indicating immune system failure.

Control

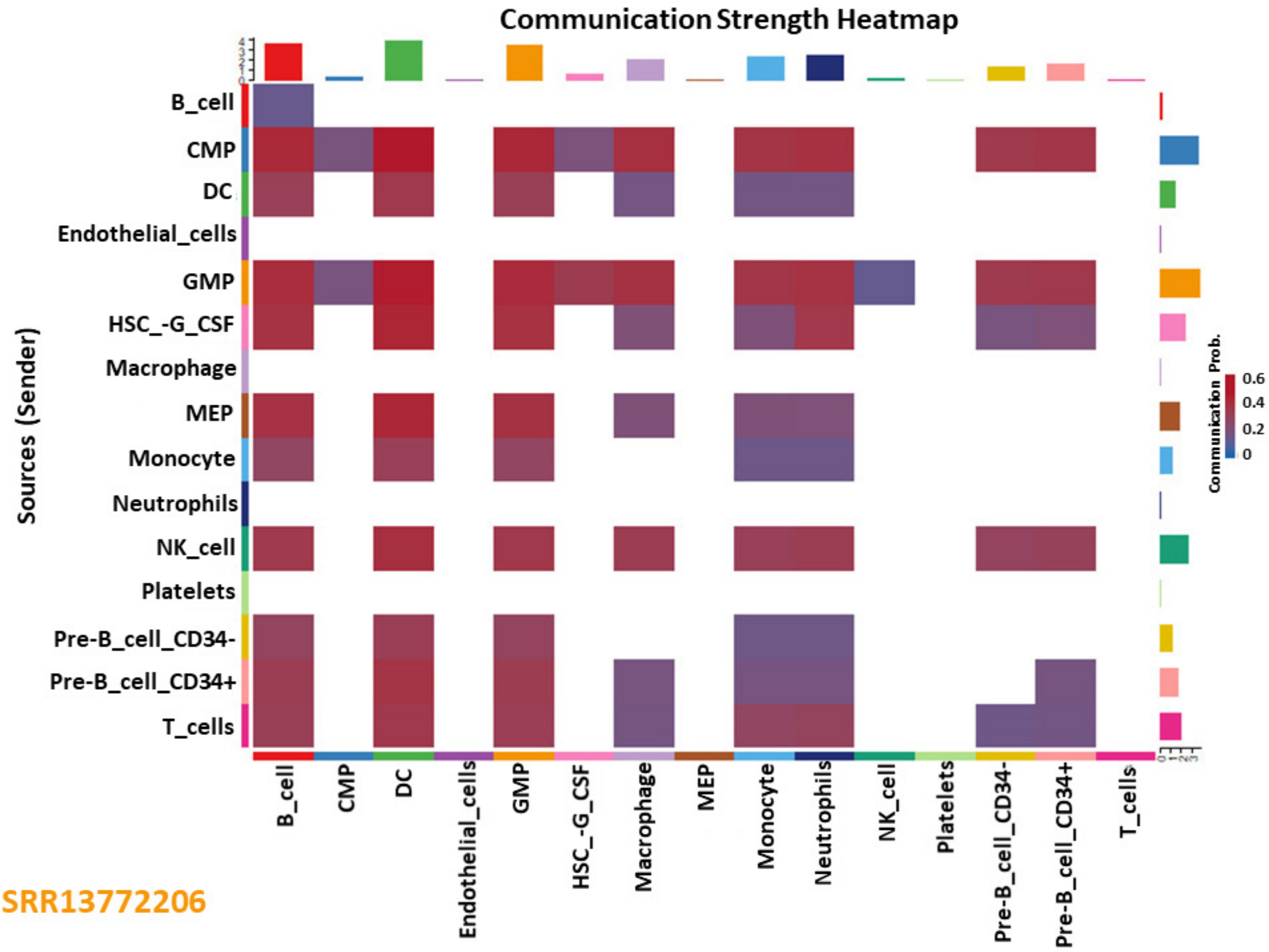

SRR13772206

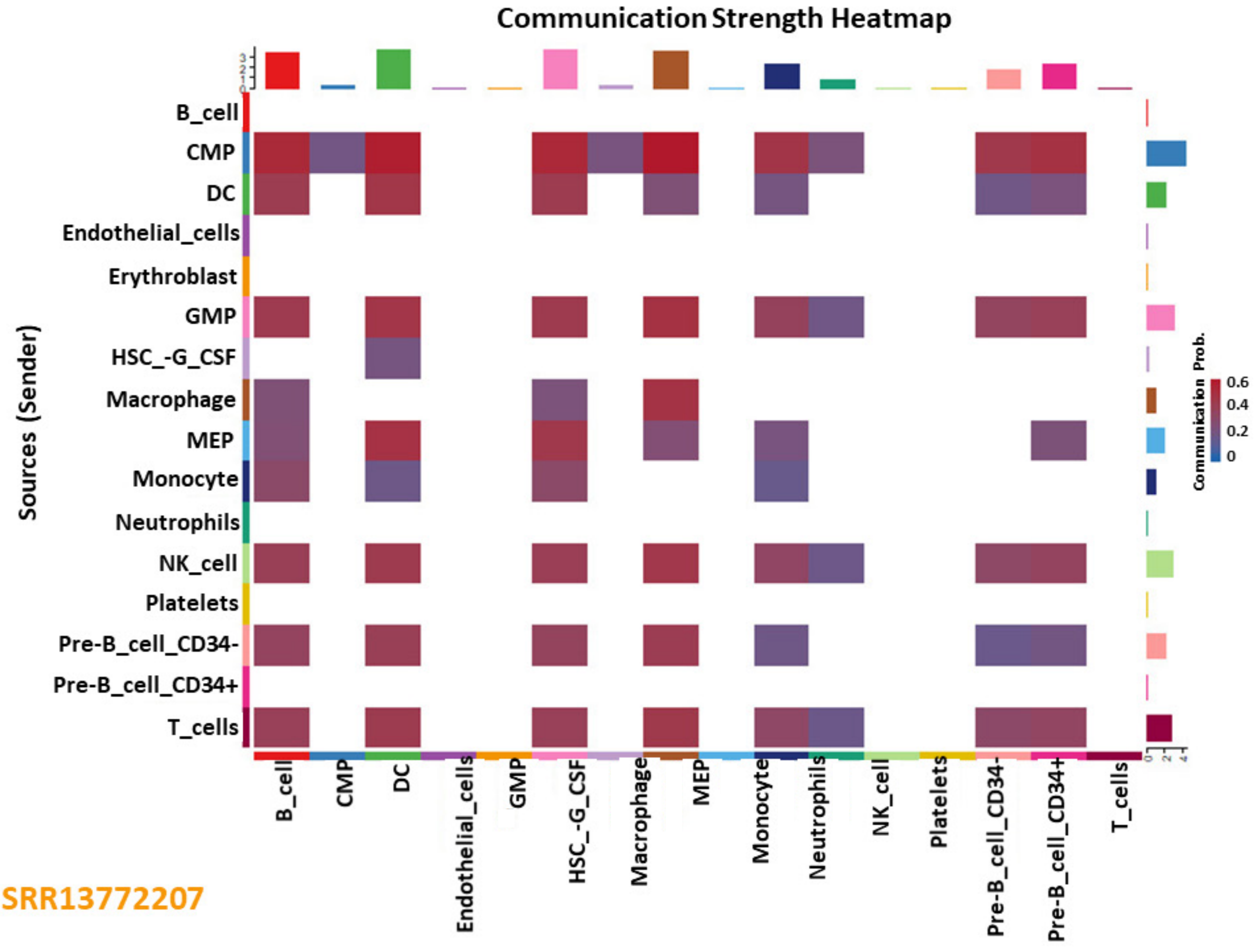

SRR13772207

Supplementary Figure S4. Heatmap representation for Control: Balanced activation of anti-inflammatory cytokines (IL-10, TGF-β) and immune surveillance markers.

Non Survived Septic Patients

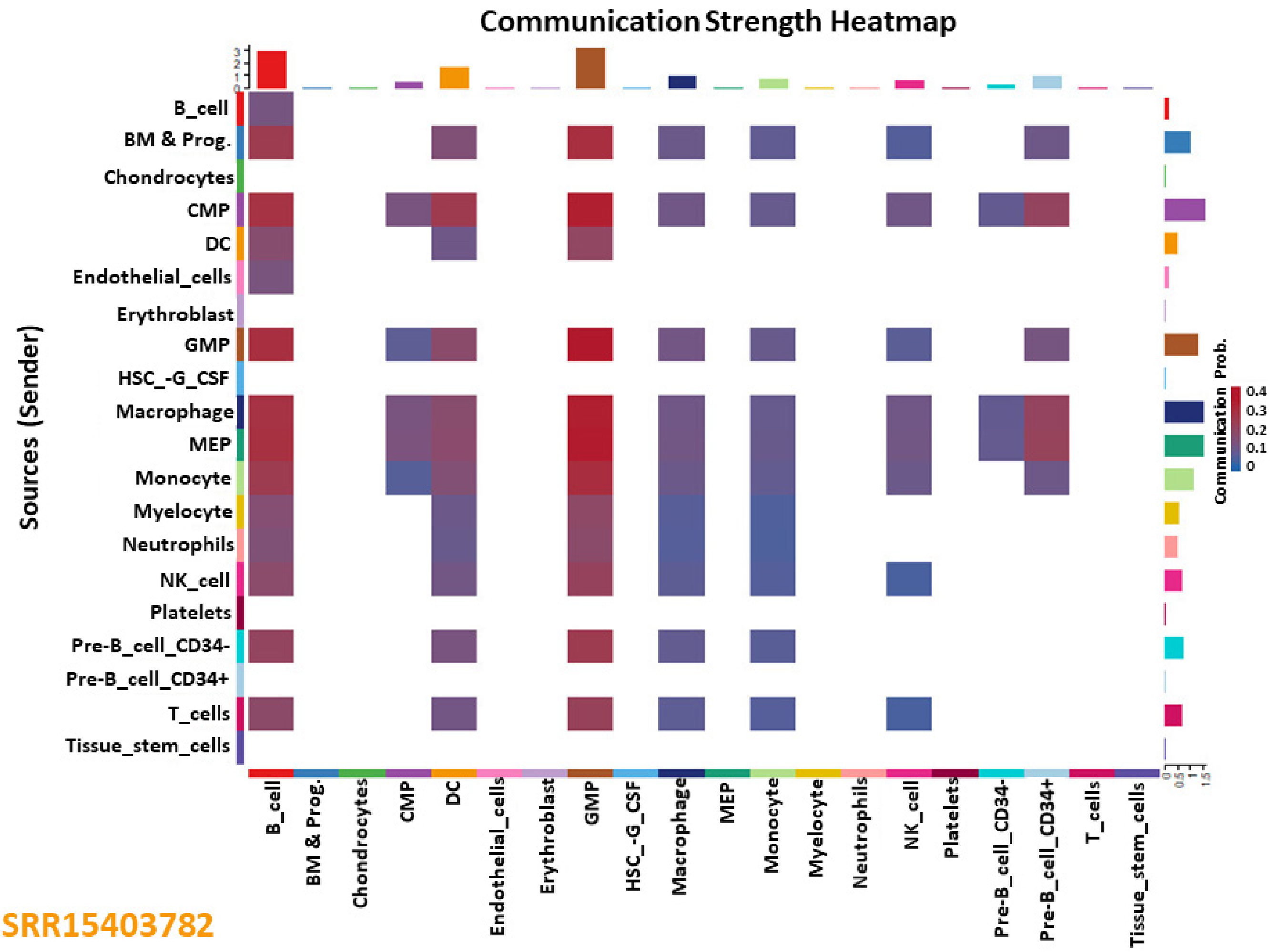

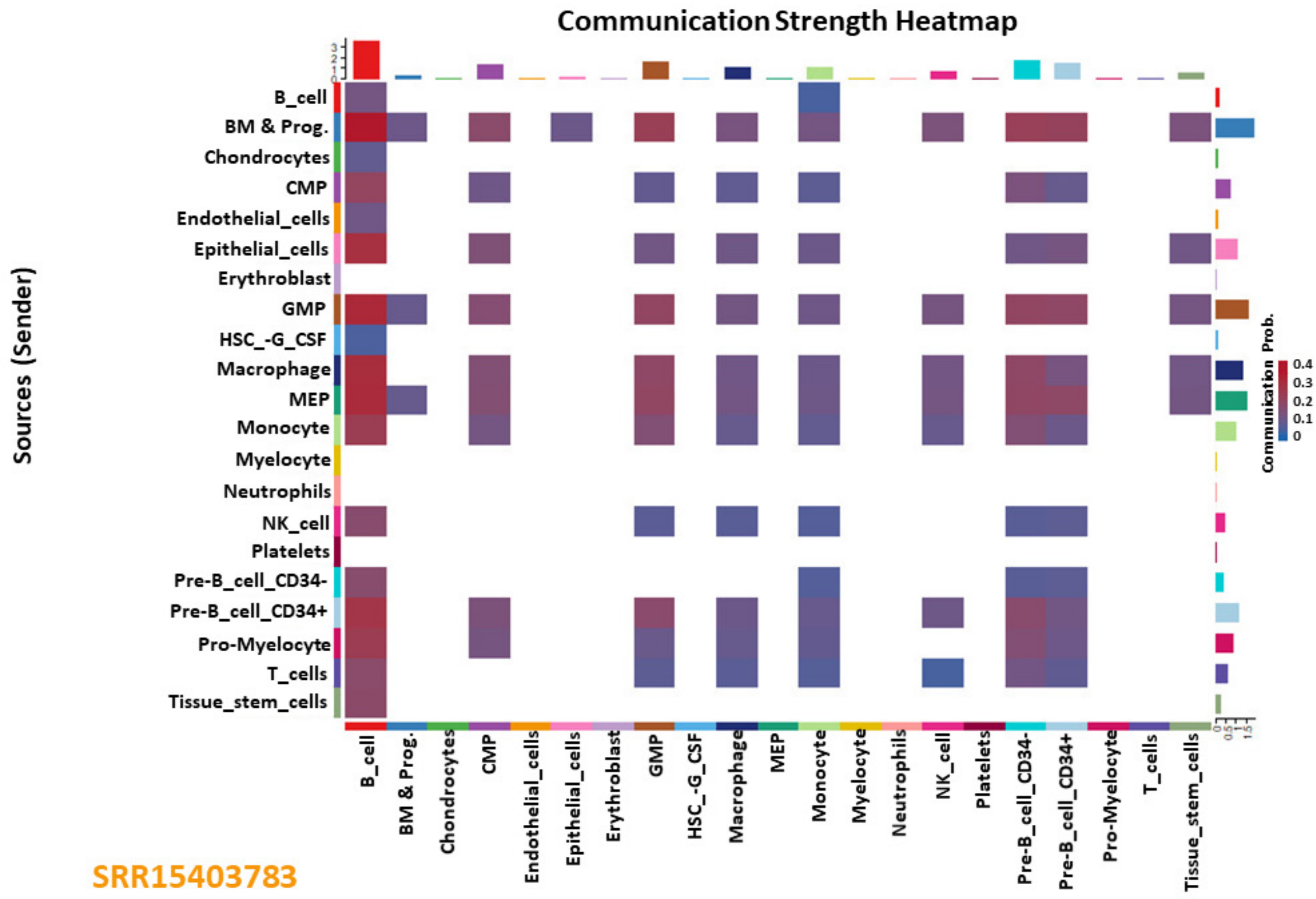

Supplementary Figure S5. Heatmap representation for Non-Survived Septic Patient: Hyperactivated neutrophil and monocyte interactions, leading to immune exhaustion and tissue damage.

Survived Septic Patients

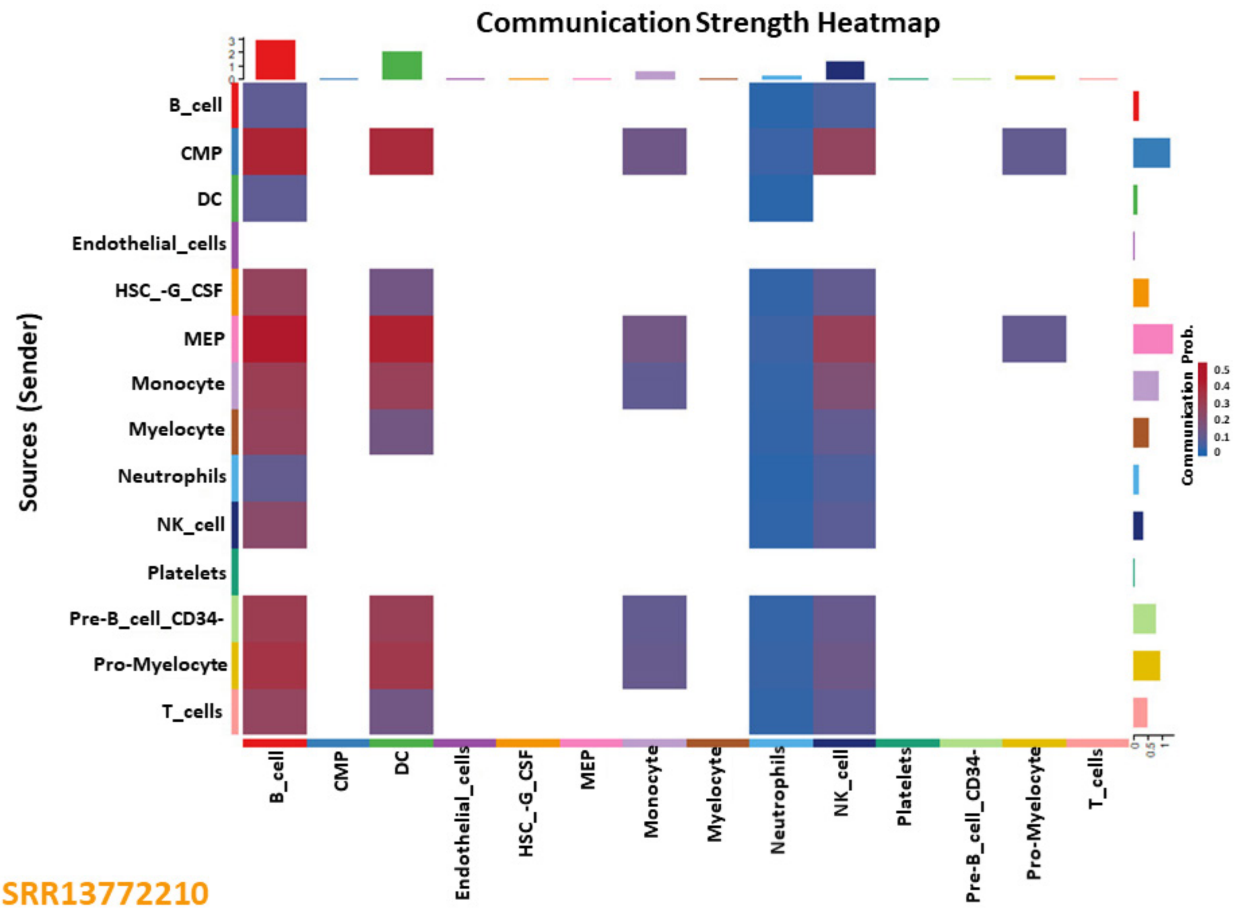

SRR13772210

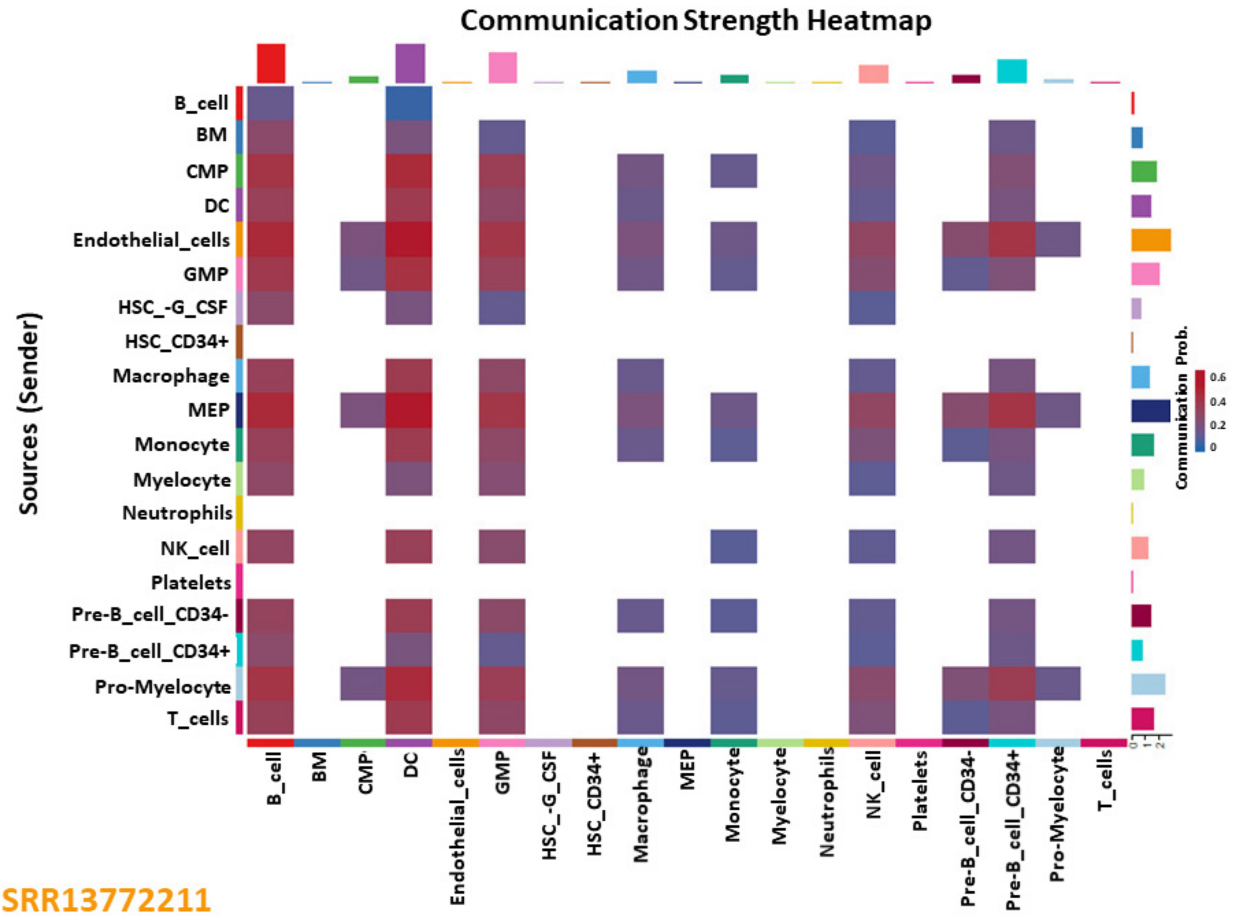

SRR13772211

Supplementary Figure S6. Heatmap representation for Survived Septic Patients: Elevated immune activation with increased expression of NF-κB, IL-6, and interferon-related genes (part 1).

Survived Septic Patients

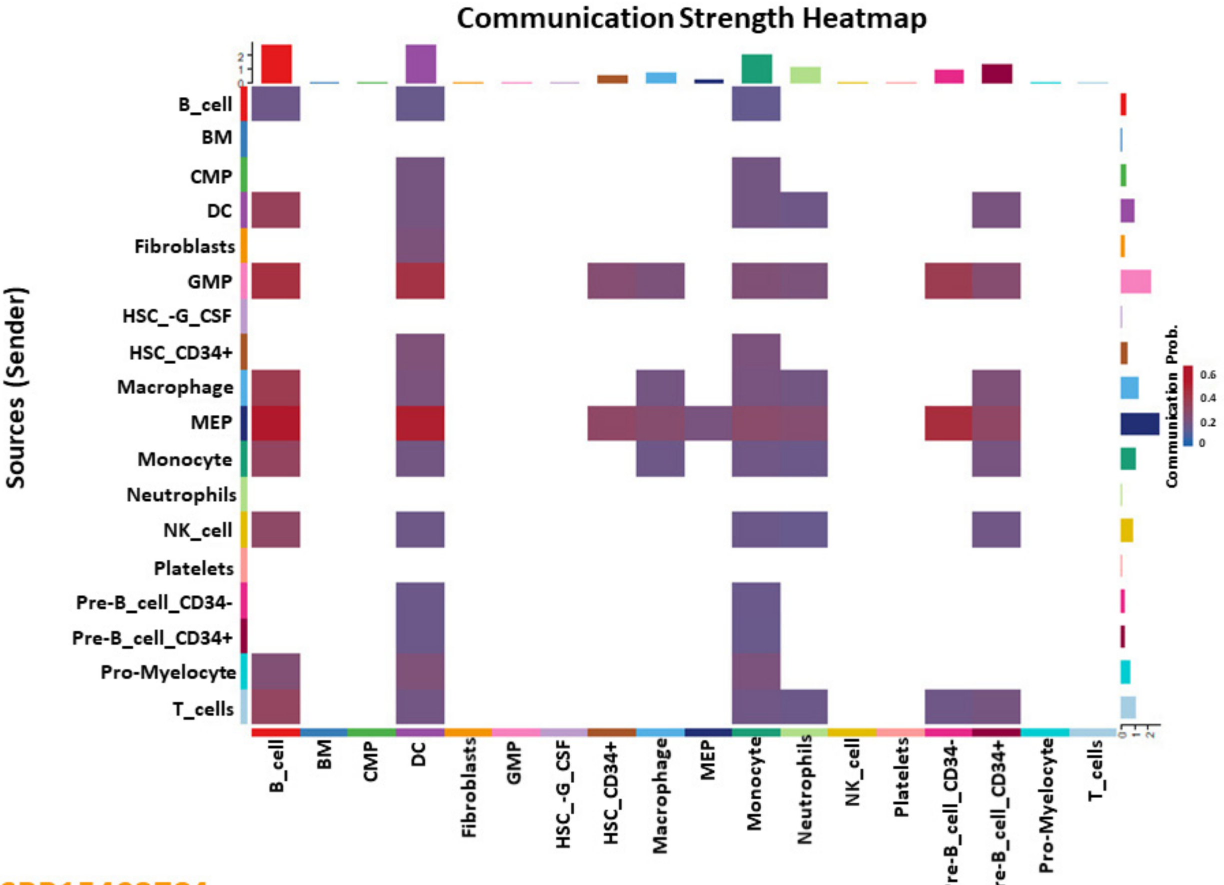

SRR15403784

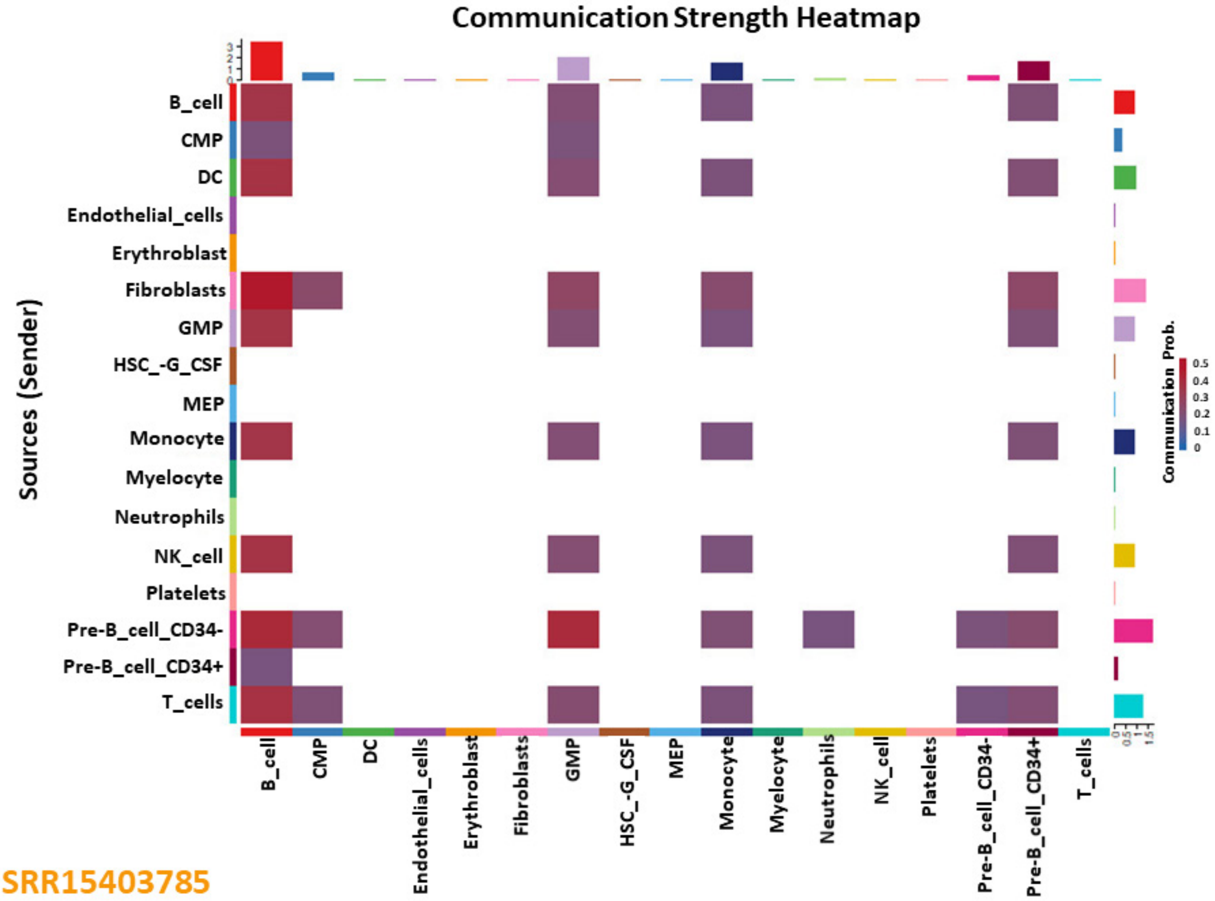

SRR15403785

Supplementary Figure S6. Heatmap representation for Survived Septic Patients: Elevated immune activation with increased expression of NF-κB, IL-6, and interferon-related genes (part 2).

Survived Septic Patients

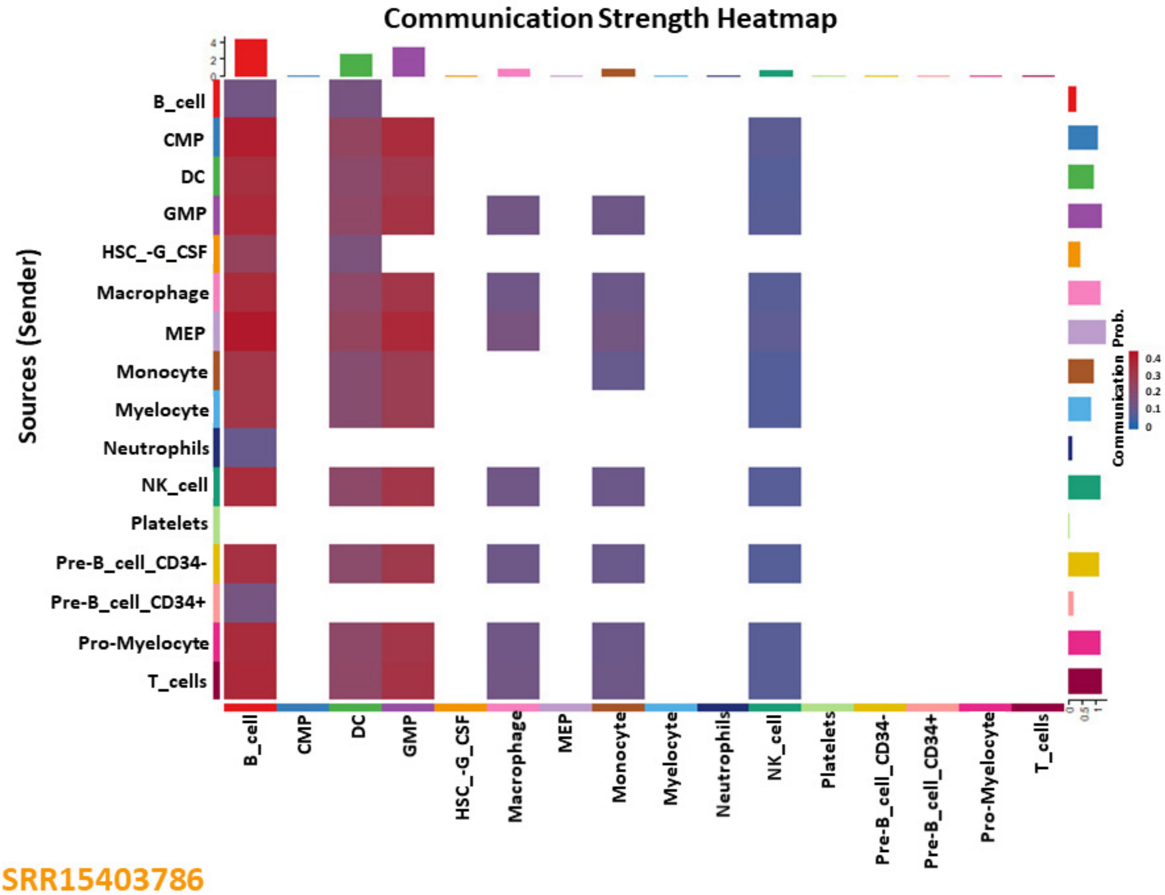

SRR15403786

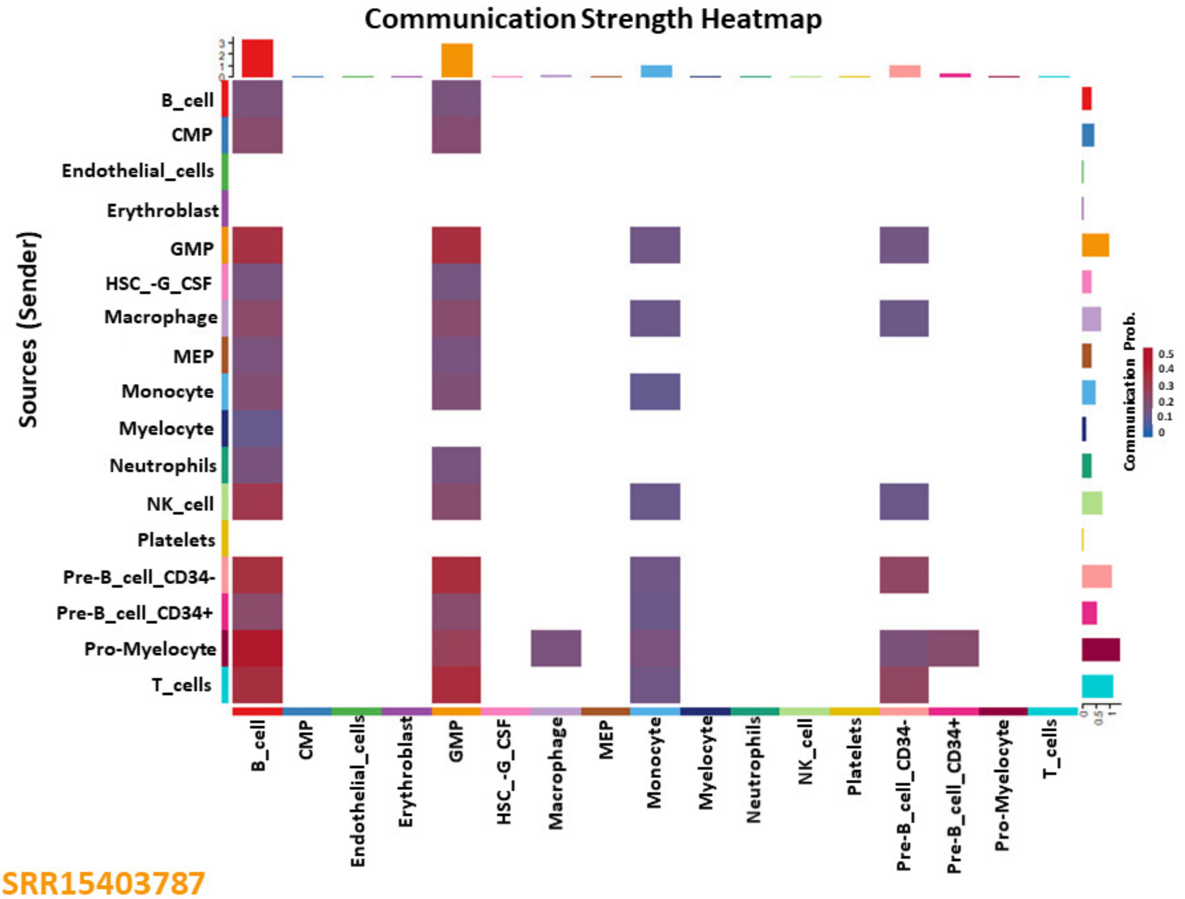

SRR15403787

Supplementary Figure S6. Heatmap representation for Survived Septic Patients: Elevated immune activation with increased expression of NF-κB, IL-6, and interferon-related genes (part 3).
