# Supplementary material for: Integrated Transcriptomic Analysis of S100A8/A9 as a Key Biomarker and Therapeutic Target in Sepsis Pathogenesis and AI Drug Repurposing
Source: Int J Mol Sci. 2025 Nov 19;26(22):11186. doi: 10.3390/ijms262211186 (PMC12653820; doi:10.3390/ijms262211186)
Supplement: Supplementary file 1 [file ijms-26-11186-s001.zip › Supplementary Figures S7-S16.pdf]

Trajectory analyses from the 200 ns MD simulation (S100-A9 Maralixibat Chloride complex)

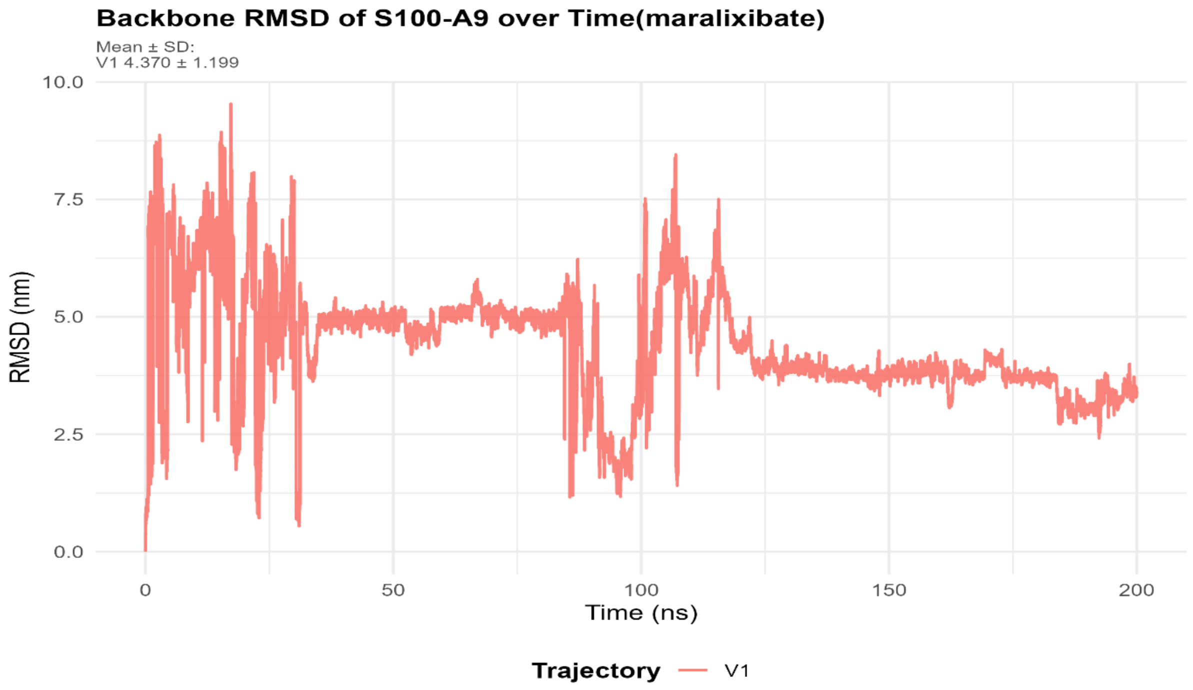

Supplementary Figure S7 - RMSD stabilization after ~10 ns

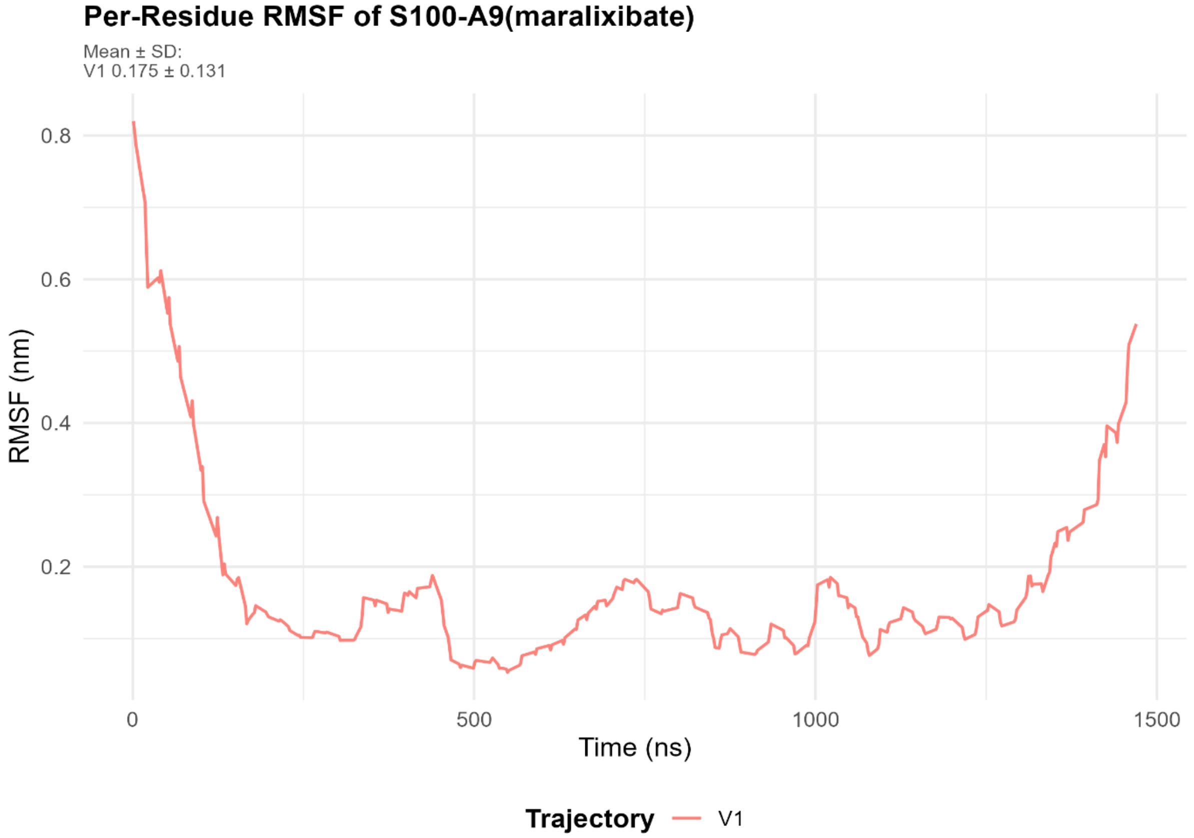

Supplementary Figure S8 -reduced residue fluctuations (RMSF) at the binding site

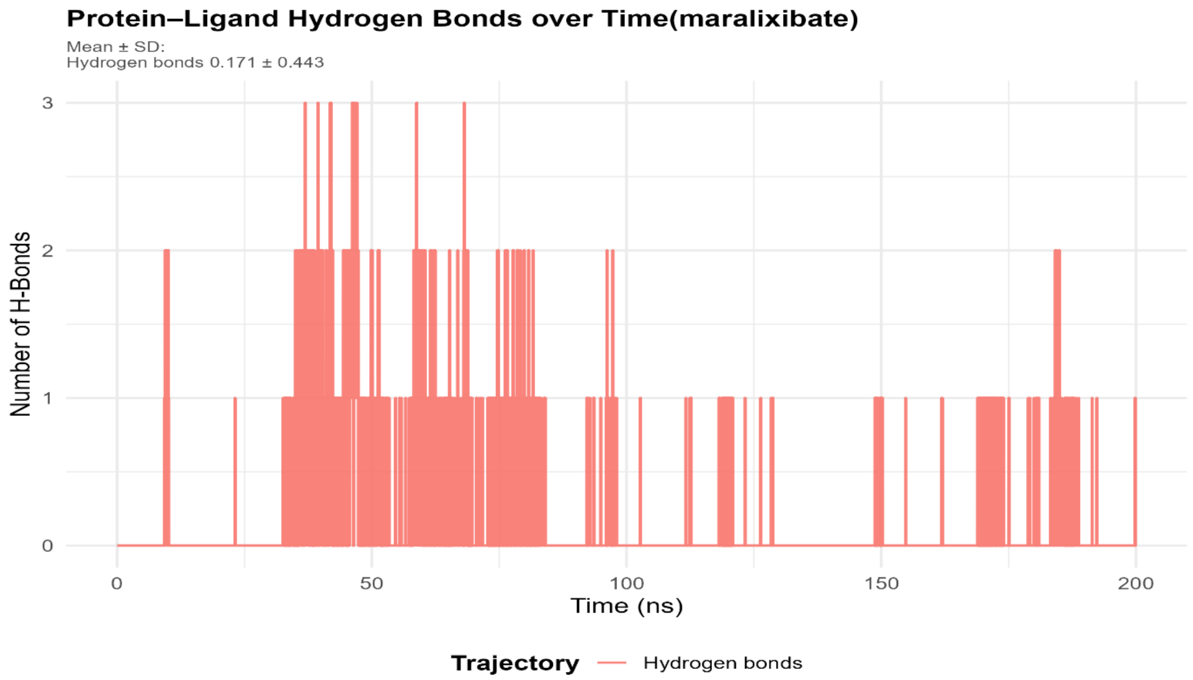

Figure S9 -2–3 persistent hydrogen bonds maintaining interaction stability

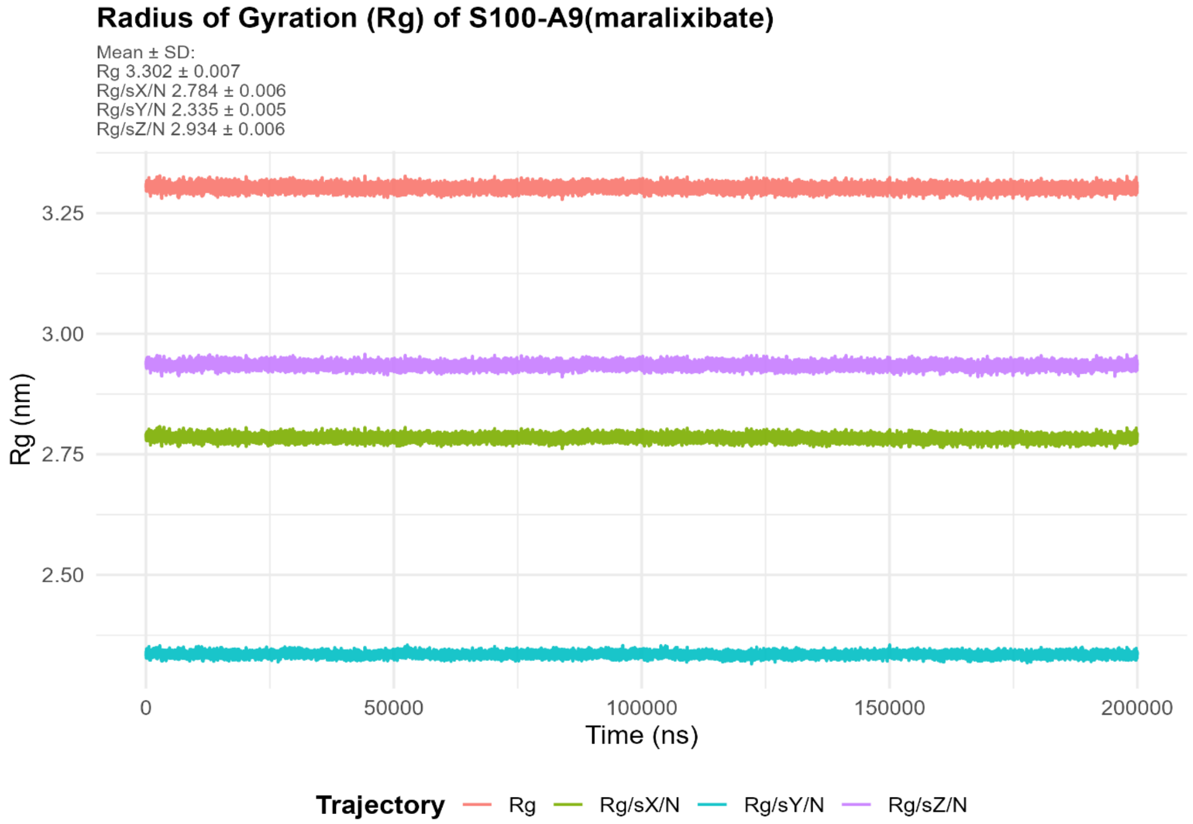

Supplementary Figure S10 - steady potential and total energy confirming system equilibration

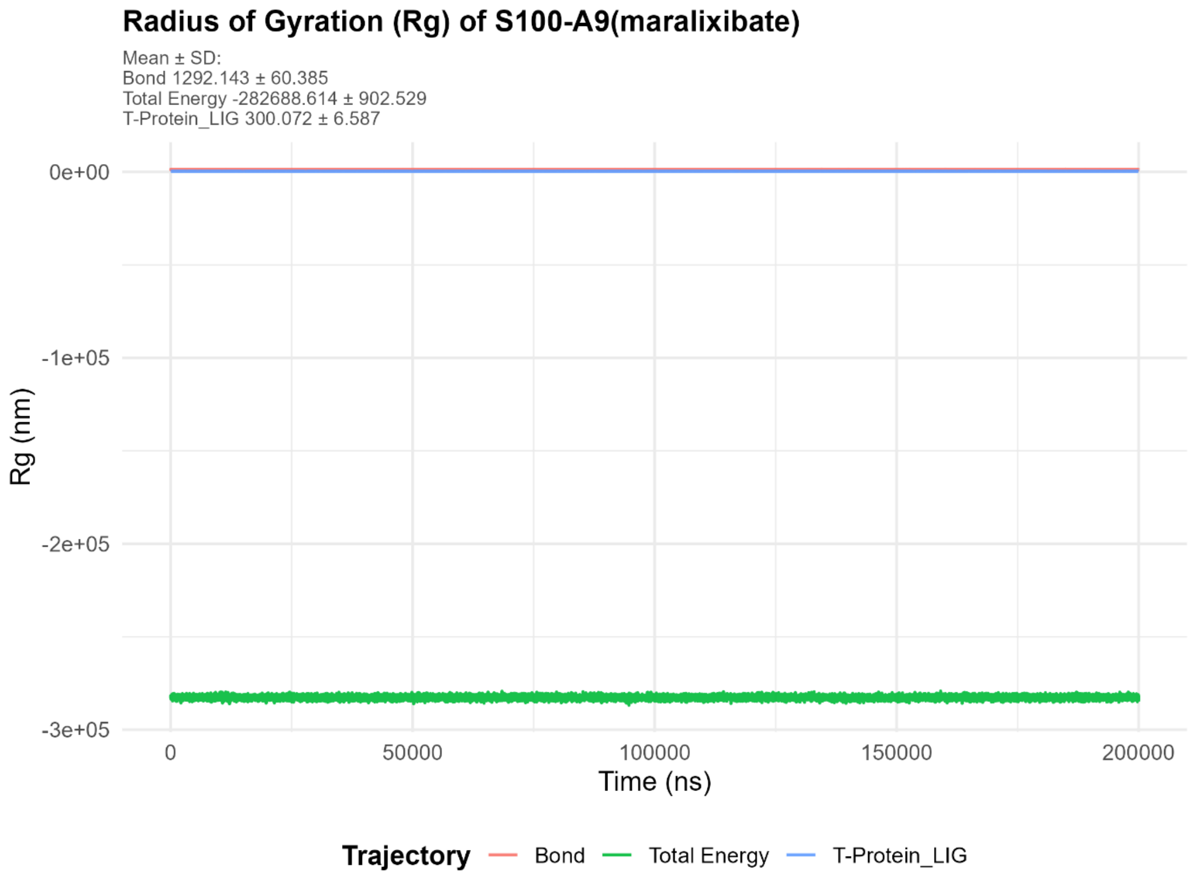

Supplementary Figure S11-consistent radius of gyration indicating structural compactness

Trajectory analyses from the 200 ns MD simulation (S100-A9–Eliquis complex)

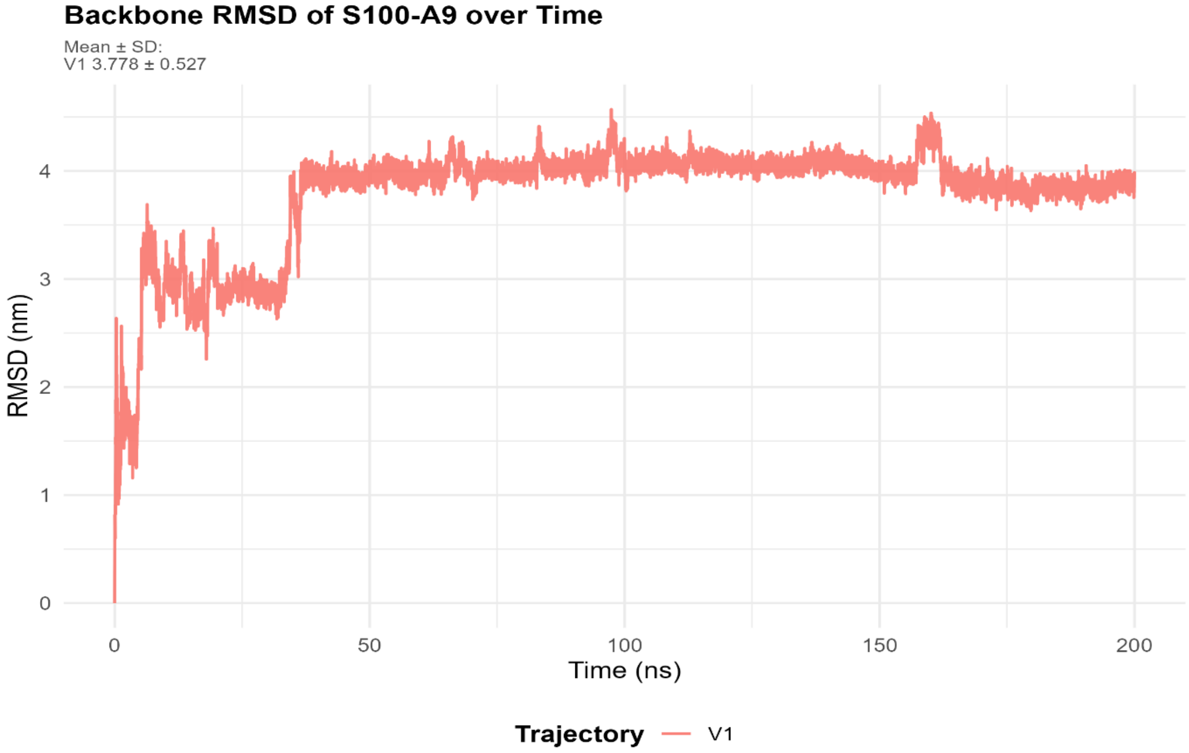

Supplementary Figure S12 - RMSD stabilization after ~10 ns,

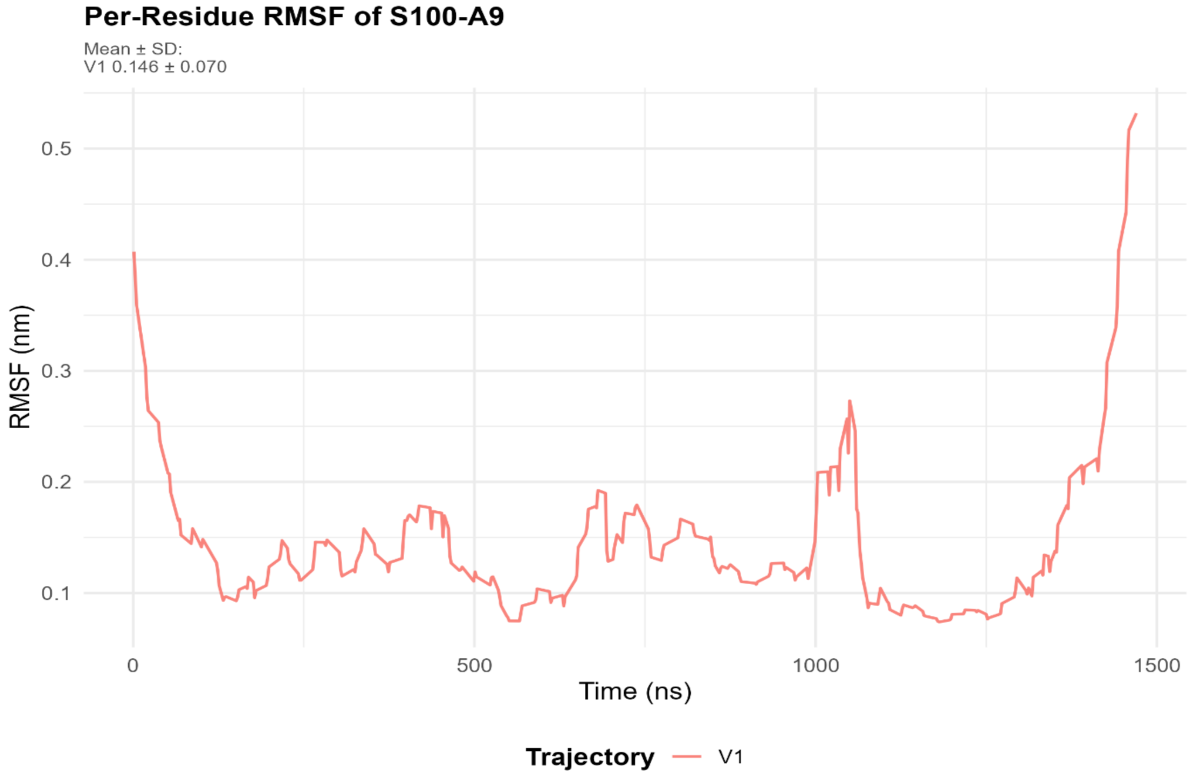

Supplementary Figure S13 -reduced residue fluctuations (RMSF) at the binding site

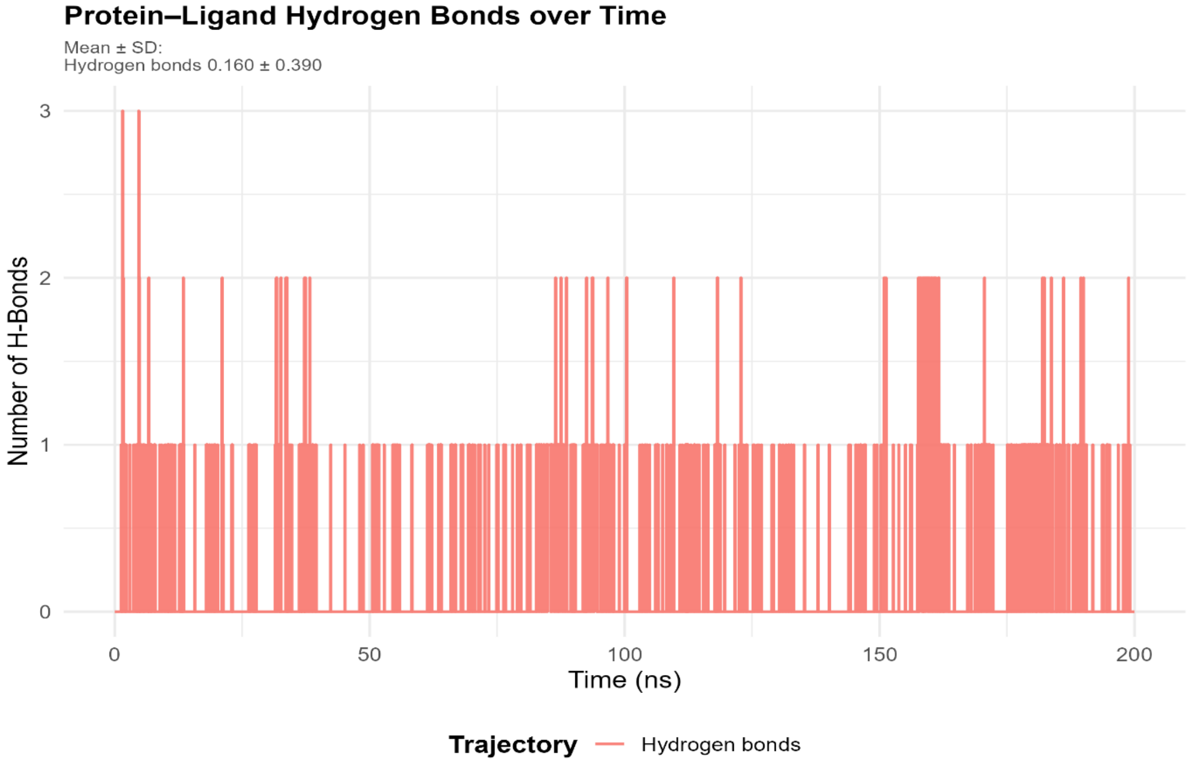

Supplementary Figure S14 -2–3 persistent hydrogen bonds maintaining interaction stability

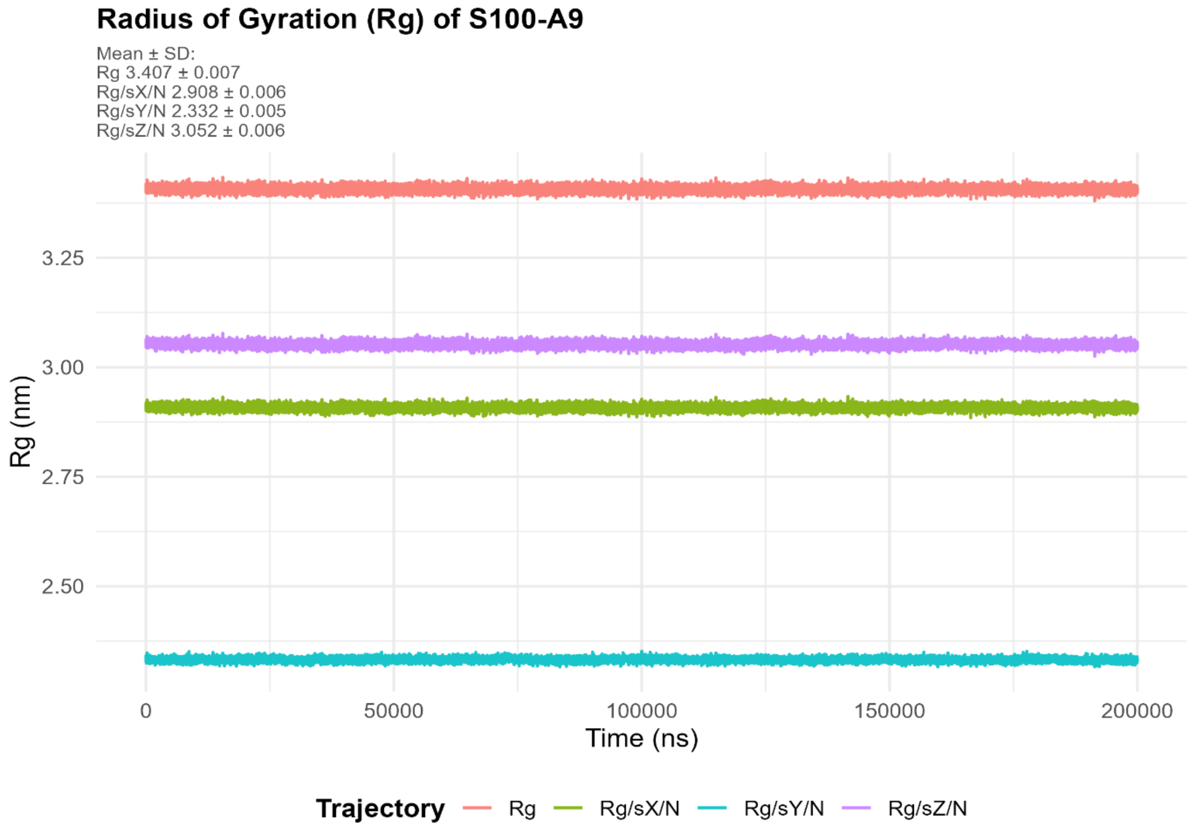

Supplementary Figure S15 - steady potential and total energy confirming system equilibration

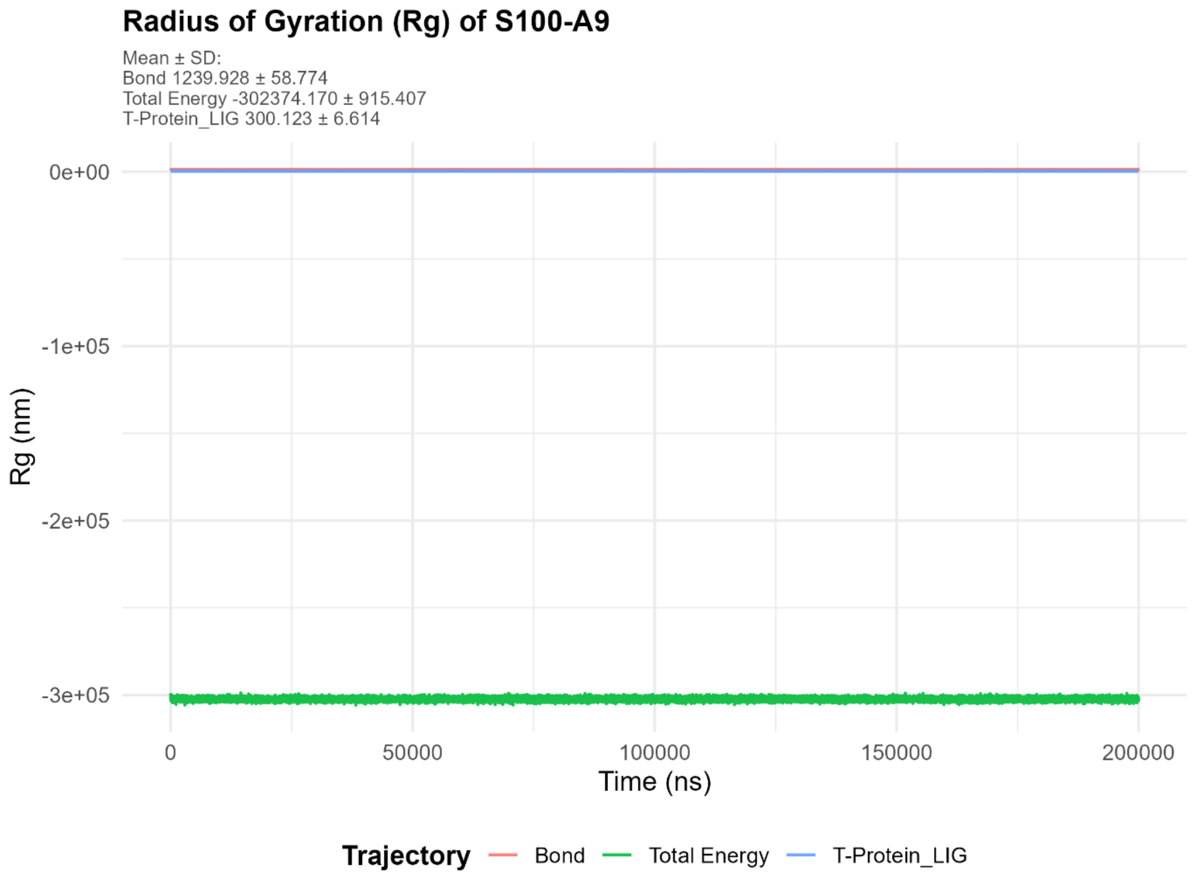

Supplementary Figure S16-consistent radius of gyration indicating structural compactness
